# Heterochromatic silencing of immune‐related genes in glia is required for BBB integrity and normal lifespan in *drosophila*

**DOI:** 10.1111/acel.13947

**Published:** 2023-08-18

**Authors:** Shunpan Shu, Mingsheng Jiang, Xue Deng, Wenkai Yue, Xu Cao, Kai Zhang, Zeqing Wang, Hao He, Jihong Cui, Qiangqiang Wang, Kun Qu, Yanshan Fang

**Affiliations:** ^1^ Interdisciplinary Research Center on Biology and Chemistry, Shanghai Institute of Organic Chemistry, Chinese Academy of Sciences Shanghai China; ^2^ University of Chinese Academy of Sciences Beijing China; ^3^ Division of Life Sciences and Medicine University of Science and Technology of China Hefei China

**Keywords:** aging, antimicrobial peptides, blood–brain barrier, gene silencing, glia, heterochromatin, IMD pathway, innate immunity

## Abstract

Glia and neurons face different challenges in aging and may engage different mechanisms to maintain their morphology and functionality. Here, we report that adult‐onset downregulation of a *Drosophila* gene *CG32529/GLAD* led to shortened lifespan and age‐dependent brain degeneration. This regulation exhibited cell type and subtype‐specificity, involving mainly surface glia (comprising the BBB) and cortex glia (wrapping neuronal soma) in flies. In accordance, pan‐glial knockdown of *GLAD* disrupted BBB integrity and the glial meshwork. *GLAD* expression in fly heads decreased with age, and the RNA‐seq analysis revealed that the most affected transcriptional changes by RNAi‐*GLAD* were associated with upregulation of immune‐related genes. Furthermore, we conducted a series of lifespan rescue experiments and the results indicated that the profound upregulation of immune and related pathways was not the consequence but cause of the degenerative phenotypes of the RNAi‐*GLAD* flies. Finally, we showed that *GLAD* encoded a heterochromatin‐associating protein that bound to the promoters of an array of immune‐related genes and kept them silenced during the cell cycle. Together, our findings demonstrate a previously unappreciated role of heterochromatic gene silencing in repressing immunity in fly glia, which is required for maintaining BBB and brain integrity as well as normal lifespan.

## Graphical abstract

Glia mediate neuroimmune and participate in blood–brain barrier (BBB). Here, we report that the fly gene *CG32528*/*GLAD* is required for normal lifespan and maintains glia, BBB and brain integrity in aging. *GLAD* functions specifically in glia, but not neurons, to suppress expression of antimicrobial peptides and other immunity‐related genes in the cell cycle via heterochromatic silencing, which suggests a previously unappreciated role and mechanism of heterochromatin in immune silencing and brain aging.

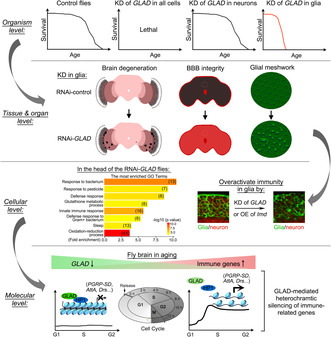

**Abbreviations**
ALG: astrocyte‐like glia
AMP: antimicrobial peptide
AttA: Attacin‐A
BAH: bromo‐adjacent homology
BBB: blood‐brain barrier
CG: cortex glia
ChIP: chromatin immunoprecipitation
CRISPR: clustered regularly interspaced palindromic repeats
Drs: drosomycin
EG: ensheathing glia
GFP: green fluorescent protein
GLAD: glia‐associated aging and degeneration
GO: gene ontology
GS: GeneSwitch
GST: glutathione S‐Transferase
H3K27me3: tri‐methylation at the K27 residue of histone H3
HP1: Heterochromatin protein 1
IFN: Interferon
IMD: immune deficiency
NF‐κB: nuclear factor κB
PG: perineurial glia
PGRP: peptidoglycan recognition protein
Rel: relish
RNAi: RNA interference
RNA‐seq: RNA sequencing
ROS: reactive oxygen species
SPG: subperineurial glia
SJ: septate junction
TJ: tight junction
TotA: Turandot A
TubGS: Tubulin‐GeneSwitch

## 1 INTRODUCTION

Aging is considered an inevitable and irreversible process of life characterized by progressive decline or alteration of many physiological functions including immune responses (Lopez‐Otin et al., 2013; Pawelec et al., 2014). On one hand, the adaptive immune functions decrease with age, leading to immunosenescence (DeVeale et al., 2004; Scheiblich et al., 2020); on the other hand, chronic elevation of the innate immunity is associated with age‐related human diseases, especially neurodegenerative disorders (Heneka et al., 2014; Labzin et al., 2018). For example, the immune and inflammatory pathways such as nuclear factor κB (NF‐κB) and interferon (IFN) signaling represent important pathomechanisms in neurodegeneration (Baruch et al., 2014; Cao et al., 2013). And, inhibition or reduction of immune and inflammatory signaling has been shown to ameliorate neurodegeneration, restore behavioral functions, and/or prolong lifespan in a variety of animal models (Baruch et al., 2014; Kounatidis et al., 2017).

Overactivation of brain immunity not only promotes neurodegeneration in disease conditions but also is associated with normal aging (DeVeale et al., 2004; Scheiblich et al., 2020). Despite of a genome‐wide upregulation of immune‐related genes with age, it is unclear whether the profound upregulation of immune‐related genes merely reflects a sum of overactivation of immune and inflammatory pathways that accumulate with age, or that these genes are coordinately regulated at the chromatin level and the mechanism controlling their expression becomes dysregulated with age.

Glia constitute more than half of the cells in the mammalian nervous system and function in many aspects including neuroimmune and neuroinflammation (Allen & Barres, 2009; Scheiblich et al., 2020). Brain aging is associated with not only deterioration of neurons but also age‐dependent alterations of glia, which also contribute to the pathogenesis of neurodegenerative diseases. Another important role of glia is the participation in the blood–brain barrier (BBB) that maintains the homeostasis and physiological function of the brain. BBB breakdown is observed in normal aging as well as in neurological disorders (Desai et al., 2007; Erickson & Banks, 2013; Montagne et al., 2015; Nation et al., 2019;). Both immune overactivation and BBB disintegration are associated with age and age‐related diseases; however, the mechanisms underlying the regulation and dysregulation of glial immunity during aging and how they impact on the BBB integrity are not well understood.

In this study, we conducted a transgenic RNA interference (RNAi) screen using the *Drosophila* model and identified several previously unknown genes involved in brain aging. Among them, the gene *CG32529/GLAD* became the focus of the current study, as its knockdown (KD) significantly reduced longevity and led to age‐dependent brain degeneration. In particular, KD of *GLAD* in glia but not neurons shortened lifespan, which caused age‐dependent glial deformation, BBB disintegration and brain degeneration due to a profound upregulation of immune‐related genes. Together, we demonstrate that GLAD keeps immune‐related genes silenced in cell cycle via heterochromatin‐mediated transcriptional repression, which is required for maintaining the glial and BBB integrity in aging and is a key lifespan determinant in *Drosophila*.

## 2 RESULTS

### 2.1 Adult‐onset downregulation of *CG32529* shortens lifespan and causes brain degeneration

This study was initiated as a part of a long‐term project in our laboratory to identify unknown genes and mechanisms involved in brain aging. Since genes required for development may also play pivotal roles in the aging process, we used the inducible GeneSwitch (GS) system to carry out a *Drosophila* transgenic RNAi screen in the adult male flies. Specifically, the *Tubulin*‐GeneSwitch (*Tub*GS) driver was used and expression of the UAS‐RNAi transgenes was induced by adding RU486 to the fly food from Day 1 after adult flies eclosed from pupae.

**FIGURE 1:**
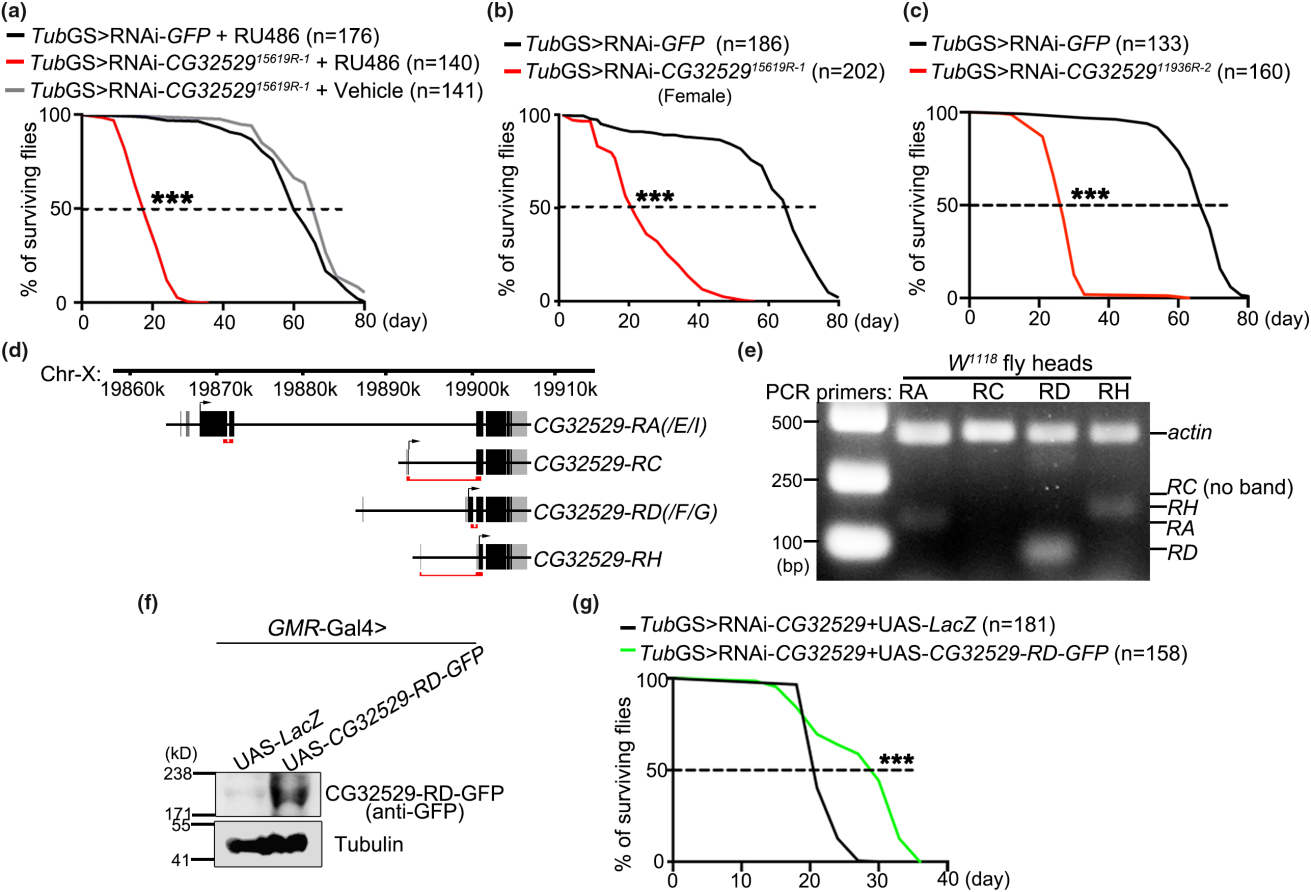
Adult‐onset downregulation of the fly gene *CG32529* leads to shortened lifespan. (a) Adult‐onset KD of the fly gene *CG32529* (with *Tub*GS) by the RNAi strain 15619R‐1 dramatically shortens the lifespan compared to the RNAi control flies (*Tub*GS > RNAi‐*GFP* + RU486) as well as the vehicle control flies (*Tub*GS > RNAi‐*CG32529* ^*15619R‐1*^ + ethanol). (b) Adult‐onset downregulation of *CG32529* (RU486 added since Day 1 of the adulthood; same the rest) in female flies (all virgins) also leads to substantially shortened lifespan, indicating that this phenotype is not gender‐specific. For ease of use, only male flies are examined in the rest lifespan assays. (c) Adult‐onset KD of *CG32529* by another RNAi strain (11936R‐2) also dramatically shortens the lifespan. (d) A schematic diagram of the *CG32529* gene in the *Drosophila* X chromosome, showing four unique coding sequences by prediction. The PCR fragments of each isoform examined in the RT‐PCR assay in (e) are indicated by the red underlines. (e) The RT‐PCR results confirm the expression of the mRNAs of *CG32529‐RA*, ‐*RD* and ‐*RH* in the adult fly heads, while *CG32529‐RD* is expressed the highest. No expression of *RC* is detected. The band of *actin* is shown as an internal control for the RT‐PCR assay. (f) Western blot analysis confirming the expression of UAS‐*CG32529‐RD‐GFP* in the fly heads (*GMR*‐Gal4). (g) The lifespan of the *Tub*GS > RNAi‐*CG32529* flies can be partially rescued by OE of UAS‐*CG32529‐RD*‐*GFP*. The log‐rank test is used for analyzing the lifespan curves and the number (*n*) of flies in each group is as indicated in the figure. ***p < 0.001. (See Table S2 for the specific genotypes of the flies tested in each figure).

In the screen, we found an RNAi line (15619R‐1) exhibited dramatically shortened lifespan compared to the RNAi control flies “*Tub*GS>RNAi‐*GFP* (green fluorescent protein)” or the vehicle control flies “*Tub*GS>RNAi‐15619R‐1 flies without RU486” (Figure 1a). RNAi‐15619R‐1 targeted the *Drosophila* gene *CG32529*. Of note, KD of *CG32529* using non‐inducible, ubiquitous drivers such as *daughterless* (*da*)‐Gal4 caused severe lethality in both male and female flies (Figure S1A,B). Since no significant gender difference was observed with *CG32529* (Figure 1b), we mainly tested male flies in the rest lifespan assays of this study. Besides, another independent RNAi fly line (11936R‐2) of *CG32529* was also lethal with ubiquitous downregulation (*da*‐Gal4) during development (Figure S1B) and short‐lived with adult‐onset KD (*Tub*GS) (Figure 1c). Since the two RNAi strains showed similar lifespan, KD efficiency and lethality phenotypes (Figure 1a–c and Figure S1B,C), for simplicity, the RNAi‐15619R‐1 strain was used in the rest of the study and referred to as the RNAi‐*CG32529* line.

The *CG32529* locus has eight transcripts annotated in the FlyBase (http://flybase.org/), with four unique coding sequences: RA (/RE/RI), RC, RD (/RF/RG), and RH (Figure 1d). We examined the expression levels of the four isoform transcripts in fly heads, and *CG32529*‐*RD* was expressed most abundantly (Figure 1e). Accordingly, we generated the UAS transgenic flies expressing this isoform fused with a GFP tag to its C‐terminus (UAS‐*CG32529‐RD*‐*GFP*) and confirmed its expression in fly heads (*GMR*‐Gal4) by western blotting (Figure 1f). Adult‐onset overexpression (OE) of *CG32529‐RD‐GFP* partially rescued the lifespan of the *Tub*GS > RNAi‐*CG32529* flies (Figure 1g), suggesting that the RD isoform played a significant role while the other isoforms might contribute additional functions. Nevertheless, these data indicate that *CG32529* is required for maintaining normal lifespan in *Drosophila*.

In order to examine the expression of *CG32529* at the protein level, an anti‐CG32529 antibody was desired. Unfortunately, several attempts to raise an anti‐CG32529 antibody all failed. We then took an alternative approach using the clustered regularly interspaced palindromic repeats (CRISPR)‐Cas9 gene‐editing system to generated a *CG32529*‐*HA* ^*knock‐in (KI)*^ fly strain, in which a 2xHA tag was inserted in‐frame with *CG32529* before the stop codon (Figure S2A). Western blot analysis of the *CG32529*‐*HA* ^*KI*^ fly heads showed multiple bands of the CG32529 protein isoforms, ranging from ~120 to ~350 kDa (possibly with some posttranslational modifications) (Figure S2B). Among them, a major band was ~150 kDa, which corresponded to the predict size of the CG32529‐RD isoform. In addition, we crossed the *Tub*GS > RNAi‐*CG32529* flies to the *CG32529‐HA* ^*KI*^ flies and examined the CG32529‐HA^KI^ protein levels in fly heads by western blotting. The results confirmed that the levels of several CG32529‐HA^KI^ protein isoforms were markedly decreased by KD of *CG32529* (the “*Tub*GS>RNAi‐*CG32529* + RU486” flies) compared to the same genotype of the flies but without the RU486 induction (Figure S2C‐S2D).

### 2.2 KD of *CG32529* / *GLAD* in glia but not neurons reduces longevity

The FlyAtlas and modENCODE data indicate that the mRNA of *CG32529* is highly expressed in the nervous system of flies. Hence, we examined the *Tub*GS > RNAi‐*CG32529* fly brains by paraffin sectioning, which revealed remarkable age‐dependent brain vacuoles (Figure S3), a common histological phenotype indicative of neurodegeneration in flies (Heisenberg & Böhl, 1979; Sunderhaus & Kretzschmar, 2016).

**FIGURE 2:**
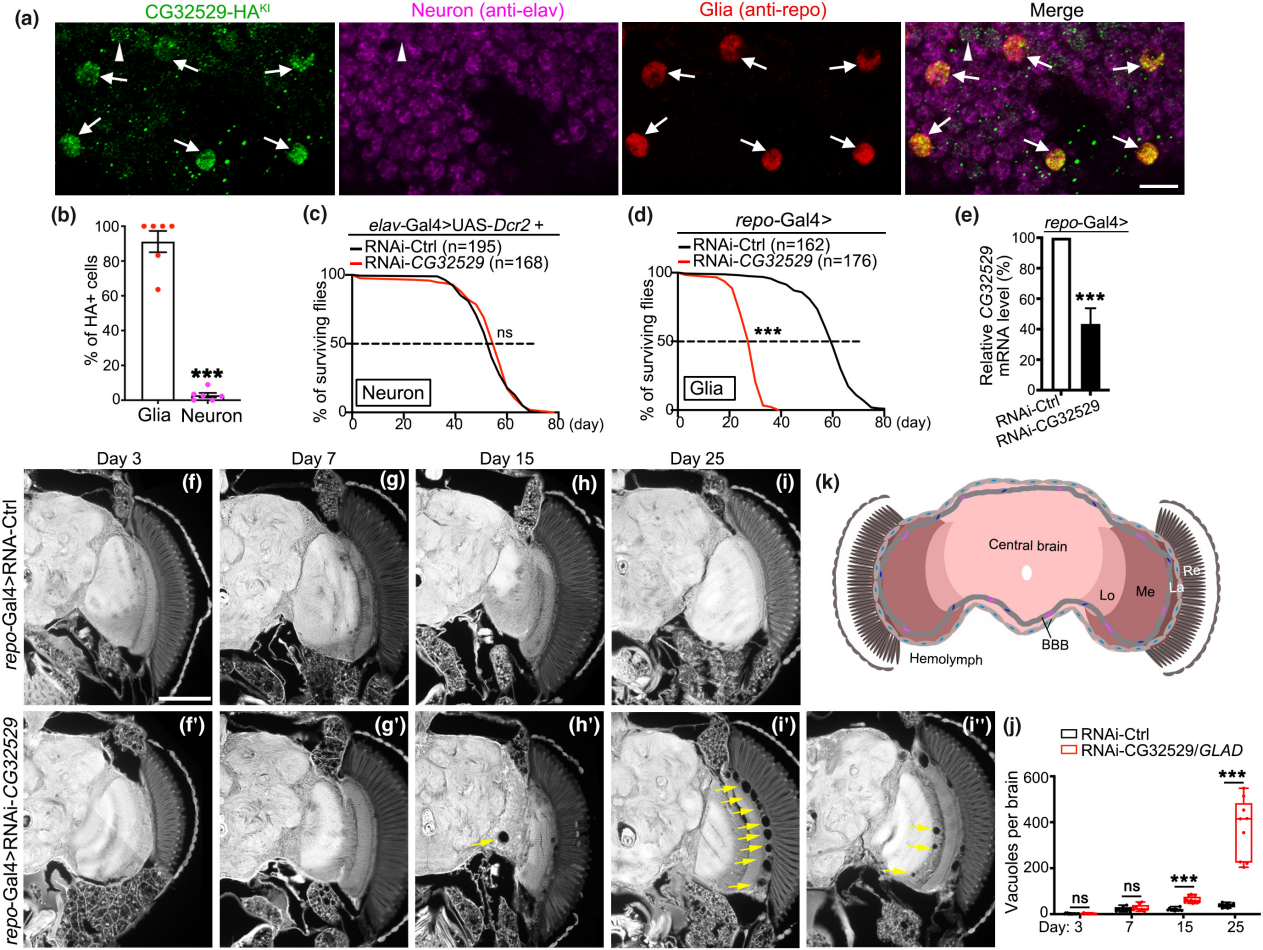
CG32529/GLAD protein is predominantly expressed in fly glia and required for normal lifespan and brain integrity. (a) Representative confocal images of the whole‐mount brains of the *CG32529*‐*HA* ^*KI*^ flies, co‐immunostained with anti‐HA for CG32529‐HA, anti‐elav for neurons, and anti‐repo for glia. Arrows, cells co‐expressing *CG32529‐HA* ^*KI*^ and repo; arrowhead, cells co‐expressing *CG32529‐HA* ^*KI*^ and elav. (b) Quantification of the percentage of glia or neurons expressing *CG32529‐HA* ^*KI*^ in an entire whole‐mount fly brain. (c, d) The lifespan of the flies with *CG32529* downregulated specifically in neurons (*elav*‐Gal4) (c) or glia (*repo*‐Gal4) (d). (e) The qPCR analysis of the mRNA levels of *CG32529* in the heads of the *repo*‐Gal4 > RNAi‐*CG32529* flies. (f–i″) Representative images of the paraffin sections of the fly brains of the *repo*‐Gal4 > RNAi‐Ctrl (f–i) or *repo*‐Gal4 > RNAi‐*GLAD* (f′–i″) at indicated ages. Arrows, brain vacuoles. (I"), a different z‐section of the same fly brain in (i′) showing vacuoles in additional brain regions. (j) Average counts of total brain vacuoles per fly brain (~30 sections/brain) at indicated ages in (f–m'). (k) A diagram showing the different regions of the fly brain and its anatomical relationship to the BBB and hemolymph. Re, retina; La, lamina; Me, medulla. Lo, the lobula complex; CB, central brain. RNAi‐Ctrl, RNAi‐*GFP*. Data are presented as mean ± SEM in (b, e) and boxplots with each data point shown in (j); *n* = 6 fly brains/group in (b), n of flies tested in each group is as indicated in (c, d), *n* = 4 in (e), and *n* = 9–15 fly brains/group in (j). The statistical significance is determined by Student's *t*‐test in (b, e and j) and log‐rank test in (c, d). ***p < 0.001; ns, not significant. Scale bars: 5 μm in (a) and 100 μm in (f–i").

To further characterize the spatial expression pattern of CG32529 in the fly brain, we used the *CG32529*‐*HA* ^*KI*^ flies we generated and did co‐immunostaining of the whole‐mount brains with anti‐HA (for CG32529‐HA^KI^), anti‐elav (for neurons) and anti‐repo (for glia) (Figure 2a). Over 90% of the repo^+^ cells were immunopositive for CG32529‐HA^KI^, whereas less than 5% of neurons (elav^+^ cells) expressed CG323529‐HA^KI^ (Figure 2b), indicating that the CG32529 protein was primarily expressed in glial cells of the fly brain. In line with the expression pattern, cell type‐specific KD of *CG32529* in neurons (*elav*‐Gal4, even with an extra copy of UAS‐*Dcr2* to boost the RNAi KD efficiency (Ni et al., 2008)) did not significantly affect longevity, whereas glia‐specific (*repo*‐Gal4) KD of *CG32529* drastically shortened the lifespan (Figure 2c–e). Furthermore, massive brain vacuoles were detected in aged *repo*‐Gal4 > RNAi‐*CG32529* flies, indicating an age‐dependent brain degeneration (Figure 2f–k). Together, because of these staggering glia‐associated aging and degeneration phenotypes, we named this fly gene “*GLAD*”.

Of note, although *GLAD* was predominantly expressed and functioned in glial cells in the fly brain, it was expressed elsewhere outside the nervous system according to FlyAtlas and modENCODE. This raised the question how much the glial function of *GLAD* contributed to the overall longevity of an organism. To address this question, we sought to determine to what extent glia‐specific OE of *GLAD* could rescue the shortened lifespan of the ubiquitous KD of *GLAD* in the *Tub*GS > RNAi‐*GLAD* flies. In order to KD and OE *GLAD* in different cells simultaneously and independently, we utilized a second binary transcription system “LexA::GAD/LexAop” together with the “GS/UAS” system. As briefly illustrated in Figure S4, the UAS‐RNAi‐*GLAD* was expressed by the ubiquitous *Tub*GS driver induced with RU486 in the adulthood (Figure S4A). To rescue *GLAD* in glia at the same time, we generated a LexAop‐*GLAD‐RD‐GFP* fly strain and drove its expression in glia using the *repo*‐LexA::GAD driver (Figure S4B). Co‐immunostaining of the whole‐mount brains confirmed the expression of GLAD‐RD‐GFP in the glia of the “*Tub*GS>RNAi‐*GLAD* + *repo*‐LexA::GAD>LexAop‐*GLAD‐RD‐GFP*” flies (Figure S4C). More importantly, the lifespan of the above flies was markedly extended compared to the flies overexpressing an unrelated LexAop‐*Luciferase* transgene in glia (the “*TubGS*>RNAi‐*GLAD* + *repo*‐LexA::GAD>LexAop‐*Luciferase*” control flies) (Figure S4D). Thus, glia‐specific OE of *GLAD* partially but significantly rescued the *TubGS* > RNAi‐*GLAD* flies, indicating that the glial function of *GLAD* contributed a significant portion to its overall impact on longevity.

### 2.3 The function of *GLAD* exhibits the glial‐subtype specificity

We noticed that the brain vacuoles in the “*repo*‐Gal4 > RNAi‐*GLAD*” flies were not randomly distributed but displayed certain spatial patterns (Figure 2i'–i" and 2k). This promoted us to examine whether the glial function of *GLAD* had any region or subtype specificity. Fly glia have five subtypes that share anatomical and functional features with their vertebrate counterparts (Kremer et al., 2017). As briefly illustrated in Figure S5A, perineurial glia (PG) (Figure S5B) and subperineurial glia (SPG) (Figure S5C) constitute the fly BBB, forming a sheet‐like, contiguous surface that covers the entire brain and separates it from the circulating hemolymph; cortex glia (CG) (Figure S5D) and astrocyte‐like glia (ALG) (Figure S5E) in the fly brain function as mammalian astrocytes; and ensheathing glia (EG) (Figure S5F) in the CNS and wrapping glia in the PNS encase fly axons similar to that of mammalian oligodendrocytes and Schwann cells, respectively (Awasaki et al., 2008; Kremer et al., 2017).

Next, we obtained of a collection of glia‐subtype Gal4 lines (Kremer et al., 2017; Li et al., 2017) and used a membrane‐tagged GFP reporter (UAS‐*mCD8‐GFP*) to examine their expression patterns in the fly brain. Among them, the ones whose expression pattern and specificity were confirmed (Figure S5B‐S5F) were then used in the subsequent lifespan assays (Figure S5G‐S5K). KD of *GLAD* in the BBB glia including PG (*NP6293*‐Gal4) and SPG (*moody*‐Gal4) (Figure S5G‐S5H) or in CG (*NP2222*‐Gal4) (Figure S5I) dramatically shortened the lifespan, while KD of *GLAD* in EG (*NP6520*‐Gal4) modestly reduced the lifespan (Figure S5J). In contrast, KD of *GLAD* in ALG (*NP3233*‐Gal4) did not significantly alter the lifespan (Figure S5K), despite that the GFP reporter driven by the ALG subtype‐specific driver *NP3233*‐Gal4 showed the broadest and strongest expression in the fly brain (Figure S5D). Interestingly, we noted that the brain vacuoles in the brains of pan‐glial KD flies “*repo*‐Gal4 > RNAi‐*GLAD*” mainly located along the border between the retina and the lamina (Figure 2i'), which matched the anatomy of the BBB glia (PG and SPG) that covered the brain surface (Figure S5B‐S5C). Additional vacuoles were observed in between the central brain and the lobula (Figure 2h’) and in the medulla (Figure 2i"), which accorded with the spatial organization of CG (Figure S5D) and EG (Figure S5F) in the fly brain. Together, the function of *GLAD* in maintaining normal lifespan exhibited glial‐subtype specificity, involving mainly BBB glia and CG.

### 2.4 KD of *GLAD* in glia causes age‐associated BBB leakage and glial deterioration

**FIGURE 3:**
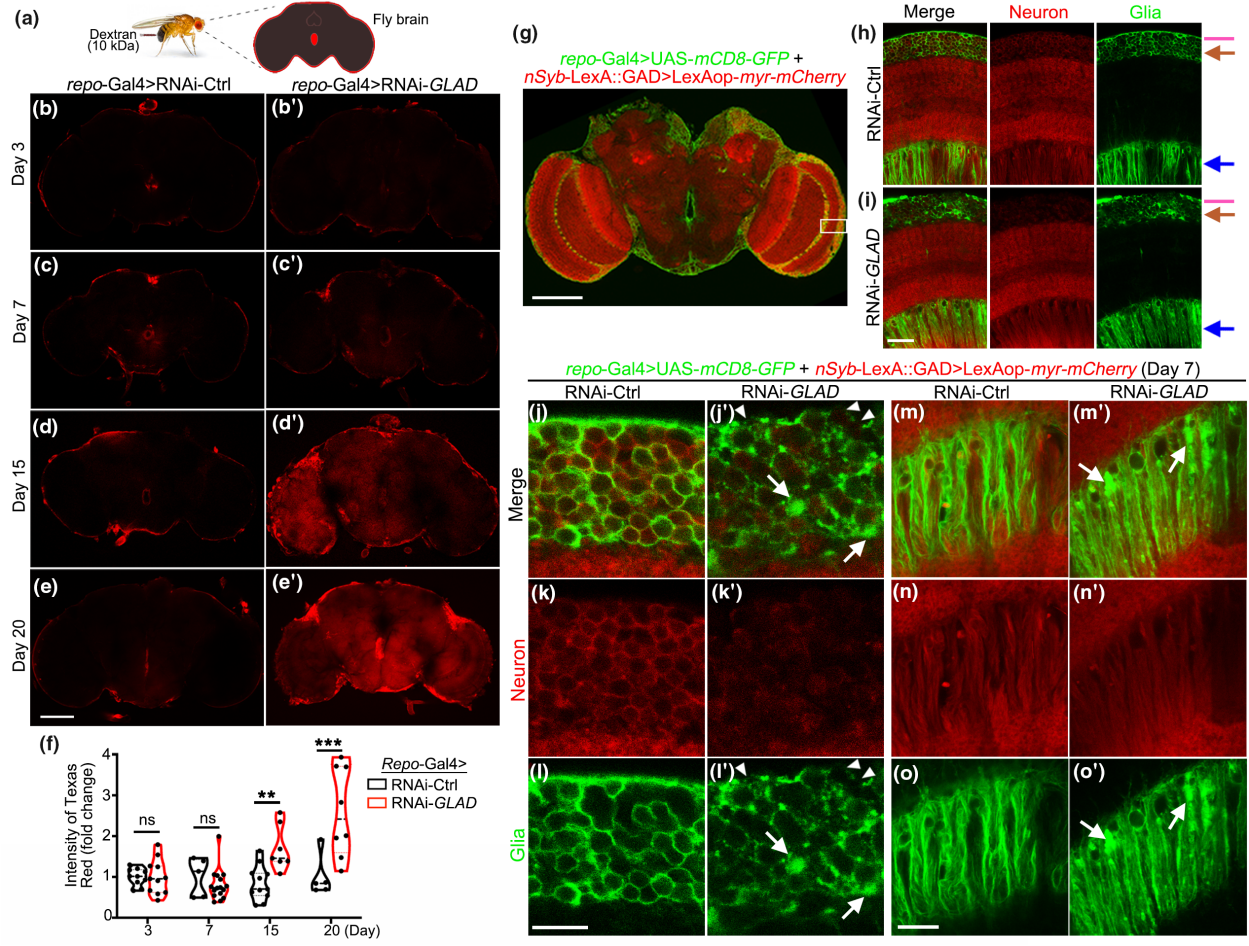
KD of *GLAD* in fly glia causes age‐associated BBB leakage and glial deformation. (a) To examine the BBB integrity, Texas Red‐labeled Dextran (10 kDa) was injected into the fly body at indicated ages and the brain is dissected for imaging ~15 h after injection. (b–f) Representative images (b–e') and quantification (f) of the relative intensity of Texas Red in the brain of the *repo*‐Gal4 > RNAi‐*GFP* (RNAi‐Ctrl) flies (b–e) or *repo*‐Gal4 > RNAi‐*GLAD* flies (b′–e') at the indicated ages. Violin plots with each data point shown; *n* = 5–17 fly brains/group. Student *t*‐test. **p < 0.01, ***p < 0.001; ns, not significant. (g) A representative image of the whole‐mount fly brain labeled with UAS‐*mCD8*‐*GFP* for glia (*repo*‐Gal4) and LexAop‐*myr‐mCherry* for neurons (*nSyb*‐lexA). The white box indicates the zoom‐in areas rotated 90‐degree counterclockwise and shown in (h, i). (h, i) Representative images of glia and neurons in the brain of the *repo*‐Gal4 > RNAi‐*Luciferase* (RNAi‐Ctrl) (h) and *repo*‐Gal4 > RNAi‐*GLAD* flies (i) on Day 7. Pink lines, the surface layer of BBB glia; brown arrows, the layer containing mostly CG and neuronal soma; blue arrows, the layer containing mostly EG and neuronal axons. (j–o') The images of (h, i) are further zoomed in to examine the morphology of BBB glia, CG and neuronal soma (j–l'), and EG and neuronal axons (m–o′). White arrows, swelling of the cytoplasm membrane of CG; white arrowheads, breakdowns of BBB glia. Scale bars: 100 μm in (b‐e', g), 25 μm in (h, i) and 10 μm in (j–o′).

BBB breakdown is associated with normal aging as well as neurodegenerative diseases (Montagne et al., 2015; Sweeney et al., 2018). The specific involvement of BBB glia in the function of *GLAD* in regulating lifespan promoted us to examine whether the BBB of the *repo*‐Gal4 > RNAi‐*GLAD* flies was integral during aging. To address this question, we conducted an in vivo BBB leakage assay in flies by injecting Texas Red‐labeled dextran (10 kDa) into the fly body (Figure 3a). The dextran circulated with the hemolymph and would not penetrate through an integral BBB (Figure 3b–e and 3b',c’). But, when the BBB was disrupted, the dextran dye leaked through, making the signal of Texas Red detectable inside the fly brain, which was observed in the brains of the *repo*‐Gal4 > RNAi‐*GLAD* flies on Day 15 and Day 20 (Figure 3d’,e'). The age‐associated BBB leakage in the *repo*‐Gal4 > RNAi‐*GLAD* flies (Figure 3b–f) indicated an essential role of *GLAD* in maintaining the BBB integrity in aging.

Fly glia not only envelope the nervous system as a whole, but also encase individual neuronal soma, dendrites and axons in the fly brain (Kremer et al., 2017) (Figure S5A, 3G‐3I). For example, CG encapsulated the neuronal soma and displayed a well‐structured, sponge‐like honeycomb morphology (Figure 3j–l). The morphology of the glial meshwork was well maintained during aging in the *repo*‐Gal4 > RNAi‐Ctrl flies (Figure S6A‐S6C and Supplemental Video S1). In contrast, the glial meshwork was deformed in the *repo*‐Gal4 > RNAi‐*GLAD* flies (Figure 3h,i, brown arrows), exhibiting swelling plasma membrane (Figure 3j’–l', white arrows; and Figure S6D‐S6F). Deformation of the glial meshwork worsened with age—by Day 15, some glia became large GFP bulges while others had degenerated, leaving huge “caverns” in the brain (Figure S6F and Supplemental Video S2). In addition, fly BBB glia showed a continuous surface covering the brain in the *repo*‐Gal4 > RNAi‐Ctrl flies but was tattered in the *repo*‐Gal4 > RNAi‐*GLAD* flies (Figure 3h,i, pink lines; and Figure 3j’–l', white arrowheads).

It was noticed that the impairment of EG (Figure 3h,i, blue arrows) was much milder than that of CG (Figure 3h,i, brown arrows) or BBB glia (Figure 3h,i, pink lines). In fact, EG displayed only minor membrane swelling (Figure 3o–o', white arrows) but no discontinuation of the cell membrane was observed. This difference was in line with the differential effects of KD of *GLAD* in the different glial subtypes on longevity (Figure S5G‐S5K). In addition, we noted that the neuronal signal labeled by the membrane‐bound LexAop‐*myr‐mCherry* (*nSyb*‐LexA::GAD) showed a marked reduction in the soma (Figure 3k–k’) but not axons (Figure 3n–n’) in the *repo*‐Gal4 > RNAi‐*GLAD* flies. Nonetheless, these data indicated that *GLAD* was required for maintaining glial and BBB integrity in the fly brain during aging.

### 2.5 Immune‐related genes are upregulated in the brain of the RNAi‐*GLAD* flies

**FIGURE 4:**
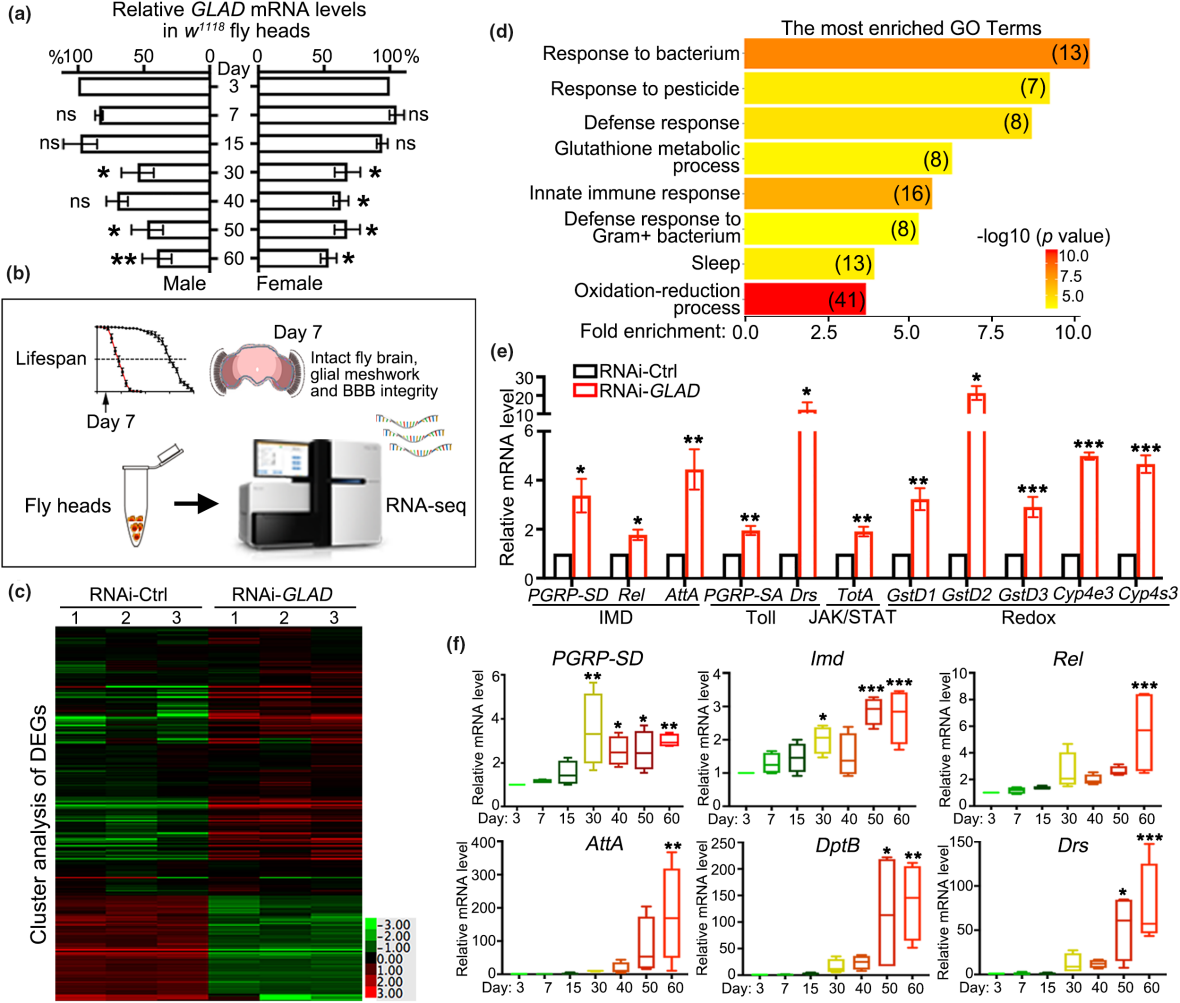
Downregulation of *GLAD* in adult fly heads causes profound upregulation of immune‐related genes. (a) The mRNA levels of *GLAD* in the heads of male and female flies (*w* ^*1118*^) at indicated ages are examined by qPCR. The mRNA level of *GLAD* on Day 3 are normalized to *actin* and set to 100%. (b) A diagram of the workflow of the RNA‐seq analysis. Fly heads are collected on Day 7 after RU486 induction, at which time point the *Tub*GS > RNAi‐*GLAD* flies have not started to die and showed marked brain degeneration, glial deformation or BBB leakage. (c) The heatmap of the 466 DEGs that are significant in all three biological repeats. *p* < 0.05 and fold change >1.5, *n* = ~200 fly heads/repeat for each genotype. (d) The most enriched GO terms of the DEGs in (c). The top eight GO terms with ‐log10 (*p*) > 3 are shown. The number of the DEGs in each GO term is shown in the parentheses. (e) The qPCR assay confirming a profound upregulation of the immune‐related genes identified in the heads of the RNA‐*GLAD* flies. (f) The qPCR examination of the mRNA levels of the representative *Drosophila* immune genes in the heads of *w* ^*1118*^ flies at the indicated ages. All mRNA levels are normalized to *actin* and set to 1 in the RNAi‐Ctrl flies (e) or in the *w* ^*1118*^ flies on Day 3 (a and f), and the relative mRNA levels in the RNAi‐*GLAD* flies (e) or flies of different ages (a and f) are shown as the fold changes. RNAi‐Ctrl, RNAi‐*GFP*. Mean ± SEM in (a, e) and boxplots in (f); *n* = 4 independent repeats of 30 fly heads in each group at each time point in (a, f), *n* = 6 in (e). One‐way ANOVA in (a, f) and Student's *t*‐test in (e). *p < 0.05, **p < 0.01, ***p < 0.001; ns, not significant.

Interestingly, when examining the mRNA levels of *GLAD* in wild‐type (WT) flies at different ages, we found that *GLAD* expression in fly heads decreased with age in both male and female animals (Figure 4a). Given that downregulation of *GLAD* in adult flies led to shortened lifespan (Figure 1a–c) and age‐dependent brain degeneration (Figure S3), the decline of *GLAD* expression in aged fly heads suggested a role of *GLAD* in brain aging. To elucidate the role and molecular mechanism of *GLAD* in the brain of adult flies, we performed the RNA sequencing (RNA‐seq) to identify differentially expressed genes (DEGs) in the *Tub*GS > RNAi‐*GLAD* flies compared to the *Tub*GS > RNAi‐*GFP* (RNAi‐Ctrl) flies (Figure 4b). Fly heads were collected on Day 7 after RU486 induction, a time point before the *TubGS* > RNAi‐*GLAD* flies started to die (Figure 1a), or showed significant brain degeneration (Figure S3), BBB leakage (Figure S6G‐S6I) or glial deformation (Figure S6J‐S6K). For the RNA‐seq analysis, we conducted three independent biological repeats of ~600 fly heads in total per genotype. Only the DEGs that consistently passed the “*p* < 0.05 and fold change > 1.5” criterion in all three repeats were included. Overall, 466 DEGs were identified (Figure 4c and Table S1).

The gene ontology (GO) term enrichment analysis revealed that the most dramatic transcriptional changes in the RNAi‐*GLAD* flies were associated with immunity, including “response to bacterium”, “response to pesticide”, “defense response”, “innate immune response” and “defense response to Gram^+^ bacterium” (Figure 4d). In addition, genes functioning in reduction–oxidation (redox) such as “glutathione metabolic process” and “oxidation–reduction process” were also enriched (Figure 4d). Of note, redox reactions and redox active molecules such as reactive oxygen species (ROS) play an important role in triggering and regulating immune responses (Gostner et al., 2013). Most of the DEGs in the RNAi‐*GLAD* flies were upregulated, which was confirmed by quantitative RT‐PCR (qPCR) (Figure 4e and Figure S7A). As mentioned above, the BBB and glia meshwork of the *Tub*GS > RNAi‐*GLAD* flies were intact at D7 (Figure S6G‐S6K), indicating that the upregulation of immune‐related genes was not merely a secondary response to BBB leakage or glial deformation. And, in line with the age‐associated decrease of *GLAD* expression (Figure 4a) and a suppressive role of *GLAD* in brain immunity (Figure 4d,e), the expression of various immune genes in the fly head was significantly increased with age (Figure 4f). Moreover, glia‐specific KD of *GLAD* (*repo*‐Gal4) also caused dramatically increased expression of immune‐related genes (Figure S7B), confirming the crucial role of *GLAD* in regulating glial cells and maintaining healthy brain immunity.

### 2.6 Downregulation of the immune deficiency (IMD) pathway and other related genes rescues the RNAi‐*GLAD* flies

To further address the question whether the profound upregulation of immune‐related genes was a cause or consequence of the shortened lifespan and degenerative phenotypes of the RNAi‐*GLAD* flies, we carried out a series of lifespan rescue experiments. In order to genetically suppress the expression of the upregulated genes in the *Tub*GS > RNAi‐*GLAD* flies, we generated a *Tub*GS > RNAi‐*GLAD* stable fly line by chromosomal recombination. Among the increased immune genes, *Relish* (*Rel*) is the *Drosophila* orthologue of the key transcription factor nuclear factor kappa B (NF‐κB) of the IMD pathway; *Attacin‐A* (*AttA*) and *Drosomycin* (*Drs*) are the effector antimicrobial peptides (AMPs) in the IMD and Toll pathways, respectively (Lemaitre & Hoffmann, 2007). *Turandot A* (*TotA*) is a downstream cytokine in the JAK/STAT pathway (Ekengren & Hultmark, 2001), which can be activated by the IMD pathway and induces further expression of immune and inflammatory genes (Kleino & Silverman, 2014). In addition, *peptidoglycan recognition protein* (*PGRP*)*‐SD* and *PGRP‐SA* were also upregulated in the RNAi‐*GLAD* flies (Figure 4e), which are extracellular pattern‐recognition receptors that can activate the IMD and Toll pathways from the upstream (Latsenko et al., 2016). Hence, we also included *Imd*, *Toll*, and several other major genes of these two immune pathways in the rescue experiments.

**FIGURE 5:**
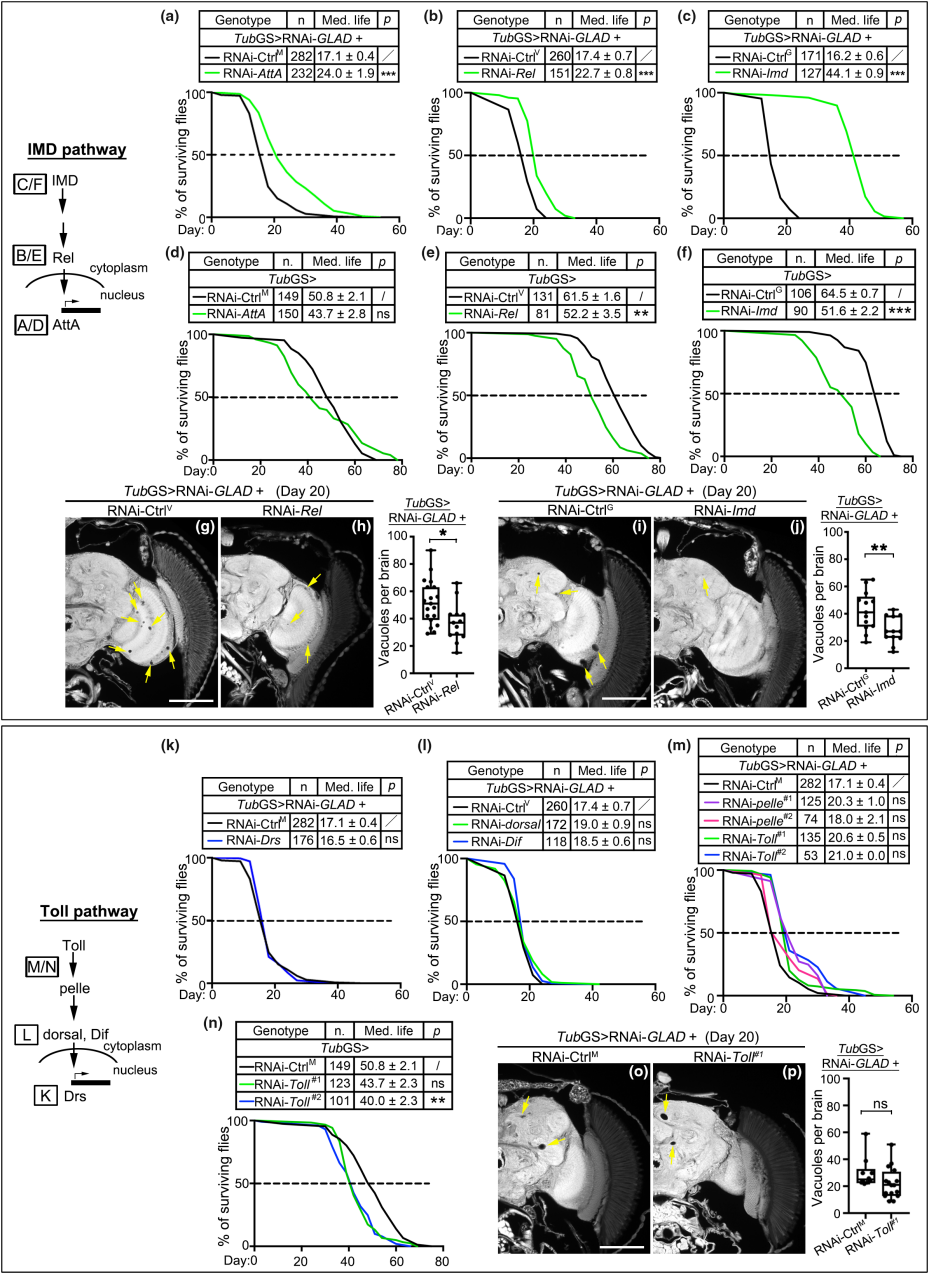
Downregulation of the IMD but not Toll immune pathway rescues the *Tub*GS > RNAi‐*GLAD* flies. (a–f) Adult‐onset KD of the IMD genes at the different step of the pathway partially rescues the shortened lifespan of the *Tub*GS > RNAi‐*GLAD* flies (a–c) without showing a general longevity‐promoting effect in the WT background (D‐F). (g–j) Representative images and quantifications of the brain vacuoles in the paraffin sections of the *Tub*GS > RNAi‐*GLAD* flies with KD of *Rel* (g, h) or *Imd* (i,j) of the IMD pathway on Day 20. (k–n) Adult‐onset KD of various Toll pathway genes cannot rescue the shortened lifespan of the *Tub*GS > RNAi‐*GLAD* flies (k–m), while the *Tub*GS > RNAi‐*Toll* flies were short‐lived (n). (o, p) Representative images and quantifications of the brain vacuoles in the paraffin sections of the “*Tub*GS>RNAi‐*GLAD* + RNAi‐*Toll*” flies on Day 20. Each RNAi line is compared to its backcrossed isogenized RNAi control line according to the origin: Ctrl^M^, RNAi‐*mCherry* (for BDSC and THFC stocks); Ctrl^V^, RNAi‐60,200 (for VDRC stocks); Ctrl^G^, RNAi‐*GFP* (for NIG‐FLY stocks). The log‐rank test is used to analyze the survival curves and the “50% survival” shown on the curve is derived from the compilation of all flies of the same genotype. n, total number of flies for each genotype, tested in multiple vials with ~20 flies per vial. Med. life, median lifespan, calculated as mean ± SEM of days when 50% or more flies in a vial had died. *p*, statistical significance of the median lifespans, determined by one‐way ANOVA. For quantifications of brain vacuoles, data are presented in boxplots with each data point shown; *n* = 10–19; Student's *t*‐test. *p < 0.05, **p < 0.01, ***p < 0.001; ns, not significant. Arrows, brain vacuoles. Scale bars: 100 μm.

We found that KD of *AttA* (Figure 5a) or *Rel* (Figure 5b) in all cells of adult flies (with *Tub*GS) modestly rescued the shortened lifespan of the RNAi‐*GLAD* flies, while KD of *Imd*, the more upstream regulator of the IMD pathway, exhibited a more robust rescue (Figure 5c). It is important to note that although suppression of the IMD pathway in some tissues such as in the gut or brain was previously shown to promote longevity in flies (Guo et al., 2014; Kounatidis et al., 2017), ubiquitous downregulation of the IMD pathway genes including *AttA*, *Re,l*, and *Imd* in adult flies did not manifest a general longevity‐promoting effect (Figure 5d–f). Rather, the lifespan of the *Tub*GS > RNAi‐*Rel* and *Tub*GS > RNAi‐*Imd* flies was even reduced (Figure 5e,f). These data not only clarified that a ubiquitous immunosuppression did not have a general longevity‐promoting effect, but also signified that the rescue of the *Tub*GS > RNAi‐*GLAD* flies by KD of the IMD pathway genes was specific. And, consistent with the effect on lifespan, KD of *Rel* (Figure 5g,h) or *Imd* (Figure 5i,j) was sufficient to rescue the brain degeneration phenotype of the aged *Tub*GS > RNAi‐*GLAD* flies.

In contrast, downregulation of the Toll pathway by KD of the downstream effector AMP *Drs* (Figure 5k), the intermediate transcription factors *dorsal* and *Dif* (Figure 5l) or the more upstream regulators *pelle* or *Toll* (Figure 5m) did not rescue the lifespan of the *Tub*GS > RNAi‐*GLAD* flies. Nevertheless, like the ubiquitous immunosuppression by KD of *Imd* (Figure 5f), adult‐onset downregulation of *Toll* in all cells did not promote longevity but rather shortened the lifespan (Figure 5n). And, consistent with the effect on lifespan, KD of *Toll* failed to rescue the brain degeneration phenotype of the aged *Tub*GS > RNAi‐*GLAD* flies (Figure 5o,p). In addition to the genes directly involved in the innate immunity, KD of the elevated cytokine gene *TotA* (Figure S8A) or the upstream genes in the JAK/STAT pathway such as *hep*, *Tab2*, *Tak1*, *ben,* and *Traf2* (Figure S8B), or KD of the upregulated redox genes such as *glutathione S‐Transferase* (*Gst*)*D1*, *GstD2*, and *Cytochrome P450‐4e3* (*Cyp4e3*) (Figure S8C) also partially rescued the lifespan the *Tub*GS > RNAi‐*GLAD* flies. And we confirmed that ubiquitous KD of these immune related genes (except for *ben*) in adult flies (*Tub*GS) did not generally extend longevity (Figure S8D‐S8F).

Together, our data demonstrated that abnormal upregulation of immune‐related genes, especially the elevation of the IMD pathway, in the *Tub*GS > RNAi‐*GLAD* flies underlay the shortened lifespan and the degenerative phenotypes of the *Tub*GS > RNAi‐*GLAD* flies.

### 2.7 Overactivation of the IMD immune pathway in fly glia induces BBB leakage and glial deterioration

Next, we sought to examine whether correcting the dysregulated levels of the IMD immune genes could rescue the BBB leakage and glial integrity in the *repo*‐Gal4 > RNAi‐*GLAD* flies. However, this has not been feasible because all attempts to generate a stable fly line carrying both the *repo*‐Gal4 and the UAS‐RNAi‐*GLAD* transgenes failed. Instead, we examined whether overactivation of the IMD pathway in fly glia would cause the BBB and/or glial disintegration as the *repo*‐Gal4 > RNAi‐*GLAD* flies.

It should be noted that although a few immune AMPs were shown to cause neurodegeneration in aged flies when overexpressed (Cao et al., 2013), significant BBB disruption or glial pathology had not been associated with immune overactivation in fly glia. Here, we found that OE of the upstream regulator *Imd* of the IMD immune pathway in glia (*repo*‐Gal4) caused remarkable brain vacuoles in aged flies; however, glial OE of *AttA* (one of the most upregulated IMD effector AMPs) was not sufficient to cause significant brain degeneration (S9A–D). More importantly, OE of *Imd* (but not *AttA*) in fly glia caused BBB leakage (Figure S9E‐S9H) and deformation of the glial meshwork (Figure S9I‐S9K) in aged flies as well as shortened lifespan (Figure S9L). Thus, our data indicated that immune overactivation in glia by either KD of *GLAD* (Figure 4) or OE of *Imd* (Figure S9) was sufficient to disrupt BBB and glial integrity in flies and substantially reduce longevity.

It is interesting to note that the failure to manifest a strong degenerative phenotype by glial OE of *AttA* suggested that the deleterious effect by glial OE of *Imd* or KD of *GLAD* on the BBB, glial and brain integrity required a profound upregulation of immune‐related genes, and it was speculated that GLAD might act in a way that masterly repressed the expression of these genes. In addition, we found that OE of *GLAD* caused developmental lethality (Figure S10A) and shortened lifespan (Figure S10B), which suggested that proper expression levels of *GLAD* and an appropriate level of immune response are critical for a normal lifespan, as abnormal immune‐overactivation and immune‐silencing are both detrimental.

### 2.8 *GLAD* encodes a *drosophila* heterochromatin‐binding protein and represses immune‐related genes in cell cycle

**FIGURE 6:**
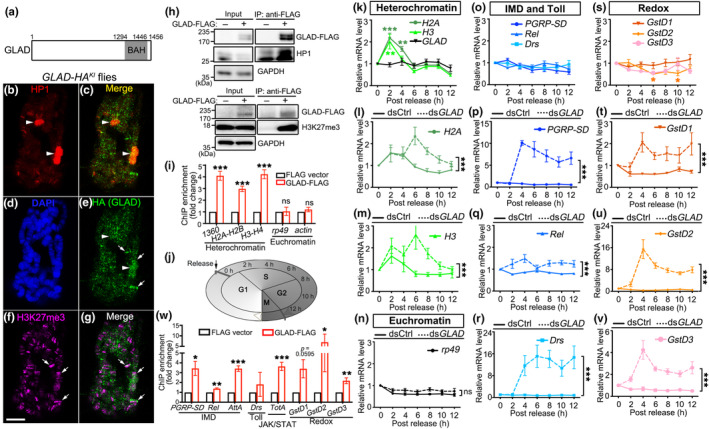
GLAD mediates heterochromatic silencing of immune‐related genes in cell cycle. (*a*) A schematic diagram of the GLAD protein containing the BAH domain. (b–g) Representative images of the polytene chromosomes of the third instar larval salivary gland cells of the *GLAD‐HA* ^*KI*^ flies, immunostained with anti‐HP1 (b), DAPI for DNA (d), anti‐HA for GLAD‐HA^KI^ (e) and anti‐H3K27me3 (f). The merged images of GLAD‐HA^KI^ with HP1a (C) or H3K27me3 (g) are also shown. Arrowheads, co‐localization of GLAD‐HA^KI^ with HP1; arrows, co‐localization of GLAD‐HA^KI^ with H3K27me3. Scale bar: 10 μm. (h) The co‐IP assays confirming the association of GLAD with HP1 (upper panel) or the histone mark H3K27me3 (lower panel) in S2R+ cells. (i) The ChIP‐qPCR analysis to evaluate the occupancy by the GLAD protein at the promoter region of the indicated genes. The ChIP enrichment is shown as the average fold change to the control vector (pIZ‐3xFLAG), indicating that GLAD specifically binds to the promoters of the heterochromatin genes (*1360*, *H2A‐H2B* and *H3‐H4*) but not those of the euchromatin genes (*rp49* and *actin*). (j) A diagraph of the cell cycle assay. S2R + cells cultured in vitro are synchronized to the G1 phase and then released. The time and the corresponding phases of the cell cycle are indicated (also see Figure S11). (k–n) Relative mRNA levels of the classical heterochromatic genes *H2A* and *H3* (k–m) and the representative euchromatic gene *rp49* (n) in the cell cycle in the absence (k) or presence (l, n) of dsRNAs. (o–v) Relative mRNA levels of the representative immune genes in the IMD (*PGRP‐SD* and *Rel*) or Toll (*Drs*) pathways (o–r) and the redox genes (*GstD1*, *D2* and *D3*) (S–V) in cell cycle assay in the absence (o, s) or presence (p–r, t–v) of dsRNAs. dsCtrl, scrambled dsRNA; ds*GLAD*, dsRNA against *GLAD*. All mRNA levels are examined by qPCR, normalized to that of *actin* and shown as relative fold changes to the basal level at 0 h (set to 1). (w) The ChIP‐qPCR assay to examine the association of GLAD to the promoters of the immune‐related genes in S2R+ cells. Mean ± SEM; *n* = 4 in (h,i, W) and *n* = 5 in (k–v). Student's *t*‐test in (i, w), one‐way ANOVA in (k, o, s), and two‐way ANOVA in (L‐N, P‐R, T‐V). *p < 0.05, **p < 0.01, ***p < 0.001; ns, not significant.

Protein domain analysis suggested that GLAD contains a bromo‐adjacent homology (BAH) domain (Figure 6a), which is found in many proteins involved in chromatin regulation and transcriptional repression (Onishi et al., 2007). In particular, BAH‐containing protein 1 (BAHD1), the putative mammalian homologue of GLAD, is a histone H3K27me3 reader and promotes heterochromatic gene silencing (Bierne et al., 2009; Zhao et al., 2016).

To examine whether GLAD was a *Drosophila* heterochromatin protein, we performed immunostaining of the fly polytene chromosomes of the third instar larval salivary gland cells of the *GLAD*‐HA^KI^ flies (Figure 6b–g). The GLAD‐HA^KI^ signal was localized to chromatin (Figure 6d,e) and enriched in the regions where heterochromatin protein 1 (HP1, a major component of the constitutive heterochromatin) (Figure 6b,c,e) or tri‐methylation at the K27 residue of histone H3 (H3K27me3, a marker of the facultative heterochromatin) (Figure 6e–g) was expressed. Further, the co‐immunoprecipitation (co‐IP) assay of *Drosophila* S2R + cells confirmed the association of GLAD with HP1 and H3K27me3 (Figure 6h). Furthermore, the chromatin immunoprecipitation (ChIP)‐qPCR assay indicated that GLAD bound to the promoters of the known fly heterochromatic genes such as *1360* and the inter‐region of the histone genes *H2A‐H2B* and *H3‐H4*, but not those of the euchromatic genes such as *rp49* and *actin* (Figure 6i).

The core histone genes *H2A* and *H3* are the classical heterochromatic genes and their expression is tightly regulated in cell cycle—synthesis of histones is required in the S phase to assemble the newly replicated DNA into chromatin, while heterochromatic silencing of *H2A* and *H3* prevents constitutive expression and excessive accumulation of histones in other phases of cell cycle (Isogai et al., 2007). To assess whether GLAD was involved in heterochromatic gene silencing, we performed the cell cycle assay in S2R + cells (Figure 6j and Figure S11A‐S11B). Indeed, the expression of *H2A* and *H3* mRNAs was increased but restricted to the S phase in the cell cycle, which reached the peak at ~2 h after released from the G1 phase and returned to the basal levels by the late S phase at ~6 h (Figure 6k). In contrast, the mRNA levels of *GLAD* as well as those immune related genes such as *PGRP‐SD*, *Rel*, *Drs*, *GstD1*, *GstD2* and *GstD3* showed no or little fluctuation in the cell cycle (Figure 6k, o and s).

Next, we downregulated *GLAD* levels in S2R + cells using double‐strand RNA (ds*GLAD*) (Figure S11C) and performed the cell cycle assay. Compared to the control cells treated with scrambled dsRNA (dsCtrl), ds*GLAD* caused *H2A* and *H3* to keep expressing beyond the S phase, reaching the peak at ~6 h and did not return to the basal level until ~12 h after releasing from the G1 phase (Figure 6l,m). In contrast, KD of *GLAD* did not increase the mRNA levels or alter the expression pattern of the euchromatic gene *rp49* in cell cycle (Figure 6n). Thus, depletion of *GLAD* led to a specific defect in heterochromatic gene silencing in fly cells.

Further examination revealed aberrant expression of immune‐related genes in the cell cycle caused by KD of *GLAD* (Figure 6o–v). The mRNA levels of the immune genes such as *PGRP‐SD*, *Rel* and *Drs* (Figure 6p–r) and the redox genes such as *GstD1*, *D2*, and *D3* (Figure 6t–v) sustained at the high levels throughout the entire cell cycle assay, which did not return to the basal levels even by the end of the G2 phase. Of note, since the basal mRNA levels of a few AMP and cytokine genes such as *AttA* and *TotA* were too low to be detected in S2R+ cells, the change in their expression during cell cycle or by KD of *GLAD* (which was measured as the relative fold change to the basal level in the qPCR assay) could not be meaningfully plotted (see *Cyp4e3* in Figure S7B for an example). Nevertheless, we performed the ChIP‐qPCR assay in S2R + cells and the results confirmed that GLAD protein bound to the promoters of the above immune‐related genes including *AttA* and *TotA* (Figure 6w), which accorded with the profound upregulation of immune‐related genes in fly heads with ubiquitous as well as glia‐specific KD of *GLAD* (Figure 4d,e and Figure S7B). Together, our study demonstrates a role of GLAD in heterochromatic silencing of immune‐related genes, which is required for maintaining glial, BBB, and brain integrity as well as normal lifespan in flies during aging.

## 3 DISCUSSION

Aging is thought to be associated with decreased immune functions (immune‐paralysis) and increased pro‐inflammatory activity (inflamm‐aging) (DeVeale et al., 2004; Scheiblich et al., 2020). Several cellular processes are considered contributing factors to the age‐related changes in immunity and inflammation. For example, (1) the adaptive immune responses decrease with age, leading to accumulation of pathogens and cellular stress, which activate inflammatory responses such as the JAK/STAT pathway (Fulop et al., 2020); (2) senescent cells accumulate in aging, which secrete a range of inflammatory cytokines, and chemokines (Pawelec et al., 2014); (3) the microbial load and pathogen diversity that one is exposed to grow with age (Clark et al., 2015); and, (4) AMPs of the IMD pathway are upregulated in the brain of aged animals even when reared in germ‐free conditions (Kounatidis et al., 2017), suggesting a pathogen‐independent mechanism underlying the age‐associated immune overactivation in the nervous system during aging.

As summarized in the Graphic Abstract, the findings of this study add a new paradigm for age‐related dysregulation of the innate immunity—the decline of heterochromatic silencing of immune‐related genes in fly glia. We show that the *Drosophila* gene *GLAD* encodes a BAH domain‐containing protein, which is localized to the heterochromatin and binds to the promotors of an array of immune‐related genes to keep them silenced. With age, *GLAD* expression and GLAD‐mediated heterochromatic silencing decrease. Meanwhile, the innate immunity including the expression of AMP genes is increased in the brain of aged flies (Figure 4f and Kounatidis et al., 2017). A progressive heterochromatin loss also led to deregulation of the genes involved in immune responses in the gut of aged flies (Chen et al., 2014). Thus, it is possible that the heterochromatin‐mediated immune silencing may be an underappreciated mechanism that is employed by other tissues and systems to keep immunity on a leash as well. In addition, it will be interesting to investigate how *GLAD* affects the cell number of glia in the fly brain in the future. This could not be done because the strong deleterious effect of RNAi‐*GLAD* made it difficult to accurately assess the cell number of glia or the effect of *GLAD* on the cell cycle in vivo in this study.

It should be pointed out that, although this work focuses on glia and immunity, GLAD does not function “only” in glia. Nor does it “only” repress immune‐related genes (see Figure S7A and Table S1). With that said, the RNA‐seq analysis of the *Tub*GS > RNAi‐*GLAD* fly heads indeed indicates that the most affected molecular pathways are enriched in immune and related functions (Figure 4d). It is interesting to note that the immune genes in the *Drosophila* genome display a significant excess of clustering in the chromatin (Wegner, 2008). And, some heterochromatin domains are enriched for genes involved in immunity in both fly and mammalian cells (Chen et al., 2014). Similarly, the genes of the *GstD* family are also clustered in the fly genome (Boutanaev et al., 2002). Heterochromatin is spatially more complex and dynamic than previously thought, and a network of subdomains can regulate diverse heterochromatin functions (Swenson et al., 2016). Thus, the chromosomal arrangement of the fly genome may facilitate the recruitment and repression of selected heterochromatin subdomains enriched for immune‐related genes. In addition, several upstream PGRP genes (e.g., *PGRP*‐*SD* and *PGRP*‐*SA*) are upregulated in the *Tub*GS > RNAi‐*GLAD* flies, which may enhance the overactivation of the IMD and Toll immune pathways, thereby further boosting the upregulation of the downstream effector AMPs.

Widespread, age‐dependent BBB disintegration is associated with physiological aging and may be an early pathological hallmark of several human diseases (Erickson & Banks, 2013; Sweeney et al., 2018; Zlokovic, 2008). In particular, the BBB is vulnerable to systemic overactivation of immune and inflammation in neurological disorders (Zlokovic, 2008). In this study, we show that local overactivation of the innate immunity in fly glia, by either KD of *GLAD* or OE of *Imd*, is sufficient to cause BBB breakdown. With the leaky BBB, immune and inflammatory factors released from glia and immune cells may leak out of the nervous system and get into the circulation system (and vice versa), which may further augment local and systemic immune and inflammatory responses to accelerate aging. Indeed, glial KD of *GLAD* or OE of *Imd* leads to age‐dependent brain degeneration and dramatically reduces longevity in flies. Of note, although the anatomy of the fly BBB is different from that of mammals, they are organized and function in a similar manner. For example, the mammalian BBB is formed by a tightly sealed monolayer of brain endothelial cells that are connected by tight junctions (TJs) (Langen et al., 2019; Limmer et al., 2014; Zlokovic, 2008); whereas in the fly BBB, the SPGs are connected to each other by septate junctions (SJs) (Limmer et al., 2014). The SJs function as the paracellular barrier like the mammalian TJs and disruption of SJs impairs the fly BBB (Babatz et al., 2018). Moreover, Claudin is a key component of the mammalian TJs and the fly SJs contains Claudin‐like molecules (Furuse & Tsukita, 2006; Limmer et al., 2014), suggesting the evolutionary conserved molecular organization of the TJs and the SJs. Therefore, it would be interesting to investigate how the GLAD‐regulated, age‐associated effector AMPs and cytokines and their mammalian counterparts affect BBB permeability in the future, which may provide us with new mechanistic insights about immune overactivation and BBB disintegration in aging and neurodegenerative diseases.

Finally, *Bahd1* is the putative mammalian homologue of *GLAD*, which encodes a core protein of the heterochromatin‐repressive complex (Bierne et al., 2009; Zhao et al., 2016). BAHD1 silences IFN‐stimulated genes and modulates innate immune defense in response to bacterial infection (Lebreton et al., 2011). The *Bahd1* null mutation causes significant perinatal death in mice, whilst the *Bahd1* ^*+/*−^ heterozygotes exhibit anxiety‐like behaviors suggesting a potential role of BAHD1 in the rodent nervous system (Pourpre et al., 2020). Future research on GLAD and BAHD1 is warranted to reveal the evolutionarily conserved and the species‐specific mechanisms in heterochromatin‐mediated immune gene silencing and how they impact on brain aging and longevity.

## AUTHOR CONTRIBUTIONS

SS, XC and YF conceived the research; SS, MJ, XD and YF designed the experiments; SS, MJ, XD, WY, ZW, JC, and QW performed the experiments; SS and XC contributed important new reagents; SS, MJ, XD, KZ, HH and KQ analyzed the data; SS, MJ, XD, KQ and YF interpreted and discussed the results; SS, MJ, XD and YF prepared the figures; and SS, MJ, XD and YF wrote the paper. All authors read and approved the final manuscript.

## CONFLICT OF INTEREST STATEMENT

The authors declare no competing interests.

## Supporting information


**Data S1:** Supporting Information

**Supplemental Table S1.** The RNA‐seq results of the 466 DEGs of the TubGS>RNAi‐GLAD flies, related to Figure 4

**Supplemental Table S2.** A summary of the genotypes of the flies examined in each figure, related to Figure 1, 2, 3, 4, 5, 6 and Figure S1‐S10

**Supplemental Video S1.** Representative 3D reconstruction of the glial meshwork (in vivo labeled by UAS‐mCD8‐GFP) of the repo‐Gal4 > RNAi‐Ctrl flies on Day 15, related to Figure 3 and Figure S6

**Supplemental Video S2.** Representative 3D reconstruction of the glial meshwork (in vivo labeled by UAS‐mCD8‐GFP) of the repo‐Gal4 > RNAi‐GLAD flies on Day 15, related to Figure 3 and Figure S6

## Data Availability

All essential data are available in the main text or the online supplemental information.
