# Supplementary material for: Heterochromatic silencing of immune‐related genes in glia is required for BBB integrity and normal lifespan in drosophila
Source: Aging Cell. 2023 Aug 18;22(10):e13947. doi: 10.1111/acel.13947 (PMC10577565; doi:10.1111/acel.13947)
Supplement: Supplementary file 1 — Data S1: Supporting Information [file ACEL-22-e13947-s002.pdf]

SUPPLEMENTAL INFORMATION

Heterochromatic silencing of immune-related genes in glia is required for BBB integrity and normal lifespan in *Drosophila*

Shunpan Shu, Mingsheng Jiang, Xue Deng, Wenkai Yue, Xu Cao, Kai Zhang, Zeqing Wang, Hao He, Jihong Cui, Qiangqiang Wang, Kun Qu, and Yanshan Fang

1. Materials and Methods

2. Supplemental Figures and Figure Legends

Figure S1, related to Figure 1

Figure S2, related to Figure 1 and 2

Figure S3, related to Figure 1 and 2

Figure S4, related to Figure 2

Figure S5, related to Figure 2 and 3

Figure S6, related to Figure 3

Figure S7, related to Figure 4

Figure S8, related to Figure 5

Figure S9, related to Figure 5

Figure S10, related to Figure 4 and 5

Figure S11, related to Figure 6

3. Supplemental Tables

Table S1, related to Figure 4

Table S2, related to Figure 1-6 and Figure S1-S10

4. Supplemental Videos

Video S1, related to Figure 3 and Figure S6

Video S2, related to Figure 3 and Figure S6

5. Supplemental References

MATERIALS AND METHODS

Drosophila strains

The following fly strains were obtained from the Bloomington *Drosophila* Stock Center (BDSC), USA: *da*-Gal4 (5460), *Actin*-Gal4 (3954), *elav*-Gal4>UAS-*Dcr2* (25750) [an extra copy of UAS-*Dcr2* is to boost the RNAi KD efficiency in fly neurons (Ni et al., 2008)], *repo*-Gal4 (7415), *repo*-LexA::GAD (67096), w^{1118} (5905), UAS-*LacZ* (8529), UAS-*mCD8-GFP* (5137), UAS-*Rel68* (55777), RNAi-*Luciferase* (31603), RNAi-*mCherry* (35785), RNAi-$Toll^{\#1}$ and RNAi-$Toll^{\#2}$ (35628, 31044), RNAi-$pelle^{\#1}$ and RNAi-$pelle^{\#2}$ (35577, 34733) and RNAi-*TotA* (58257). The following flies were from the Fly Stocks of National Institute of Genetics (NIG-FLY), Japan: RNAi-*GFP*, RNAi-CG32529 (15619R-1, 11936R-2), RNAi-*GstD1* (10045R-4), RNAi-*GstD2* (4181R-1), RNAi-*Cyp4e3* (4105R-3) and RNAi-*Imd* (5576R-2). The followings were from the Tsinghua Fly Center (THFC), China: RNAi-*Drs* (TH03169.N) and RNAi-*AttA* (TH03175.N). The followings were from the Vienna *Drosophila* Resource Center (VDRC), Austria: RNAi-control (60200), RNAi-*dorsal* (45998), RNAi-*dif* (30579), RNAi-*Traf2* (16125), RNAi-*ben* (109638), RNAi-*Tak1* (101357), RNAi-*Tab2* (100326), RNAi-*hep* (109277) and RNAi-*Rel* (49413). The glia-subtype Gal4 driver lines were from the *Drosophila* Genomics and Genetic Resources of the Kyoto Stock Center (DGRC-Kyoto), Japan: *NP6293*-Gal4 (105188), *NP2222*-Gal4 (112830), *NP6520*-Gal4 (105240) and *NP3233*-Gal4 (113173). The following lines are kind gifts from the fly community: *Moody*-Gal4 (X. Bi), *Tub*GS (N. Bonini), *nSyb*-LexA::GAD (G. Rubin), LexAop-*myr-mCherry* (M. Landgraf), *repo*-LexAop::GAD,LexAop-*mCD8-GFP* (M. Ho) and UAS-*Imd* (L. Xue).

For simultaneous and independent genetic manipulations and/or *in vivo* imaging, the following stable fly lines carrying multiple transgenes were generated by chromosomal recombination:

yw;;*Tub*GS,RNAi-*GLAD*/*TM3.Ser*

yw,repo-LexA::GAD;;LexAop-*Luciferase*/*TM3.Ser*

yw,*repo*-LexA::GAD;;LexAop-*GLAD-RD-GFP*/*TM3.Ser*

w;UAS-*mCD8-GFP*;*repo*-Gal4

yw;UAS-mCD8-*GFP*/*CyO*;*repo*-Gal4,*nSyb*-LexA::GAD,LexAop-*myr-mCherry*/*TM3.Ser*

The UAS-*GLAD-RD-GFP* transgenic fly strain was generated by *P*-element transposon in a *y'w'* background. The transgenic strains of LexAop-*Luciferase,* LexAop-*GLAD-GFP,* UAS-*AttA*-HA were generated by ΦC31 integrase-mediated, site-specific integration, and the landing site stock used was UAS-phi2b2a;VK5 (75B1). The above newly generated fly strains as well as the UAS-*lacZ* control line were backcrossed into our laboratory background w^{1118} for five generations before testing in the lifespan assays. For the various RNAi flies examined in this study, the lifespan is compared to their backcrossed isogenized RNAi control line as specified in each experiment. The specific genotypes of the flies shown in each figure are summarized in Table S2.

All flies were raised on standard cornmeal media and maintained at 25 °C and 60% relative humidity. To induce the expression of the GeneSwitch drivers such as *Tub*GS, CNS glia-GS and PNS glia-GS, flies were raised on regular fly food supplemented with 160 μg/mL RU486 (mifepristone; TCI, 84371-65-3) dissolved in ethanol.

Lifespan assays

For the lifespan experiments, 20 male flies per vial and 7-10 vials per group were tested. Flies were transferred to the fresh fly food every 3 days. The log-rank test was used for analyzing the lifespan curves and the "50% survival" shown on the curves was derived from the compilation of all vials of the same group. The actual median lifespan of each group was calculated as the average number of days at which 50% of the flies in a vial died. The statistical significance of the median lifespans between two or more groups was determined by Student's *t*-test or one-way analysis of variance (ANOVA), respectively. The flies lost prior to natural death because of escape or accidental death were excluded from the final analysis.

Plasmids and constructs

To generate the pIZ-3xFLAG plasmid, 3x*FLA*G was inserted into the pIZ-V5-6×His (Invitrogen) vector between the Xho I and Xba I sites using the ClonExpress One Step Cloning Kit (Vazyme).

For the pIZ-GLAD-FLAG plasmid, the *GLAD* cDNA was amplified from the w^{1118} fly heads by RT-PCR and inserted into the pIZ-3xFLAG vector using the Eco R I and Xho I sites.

For the pUAST-*GLAD*-GFP plasmid, the *GLAD*-GFP fragment was amplified using the above plasmid as a template and inserted into the pUAST vector using the Xho I and Xba I sites.

To generate the pBID-*AttA*-HA plasmid, the *AttA* cDNA was amplified from the w^{1118} fly heads by RT-PCR and inserted into the pBID-UASC vector using the Xho I and Xba I sites.

All constructs were confirmed by sequencing before use. The primers used in the above experiments are listed below:

*3×FLA*G-F: 5′-
TCGAGCACCATGGATTACAAGGATGATGATGATAAGGATTACAAGGATGATGATGATAA
GGATTACAAGGATGATGATGATAAGTAAT-3′

*3×FLA*G-R: 5′-
CTAGATTACTTATCATCATCATCCTTGTAATCCTTATCATCATCATCCTTGTAATCCTTAT
CATCATCATCCTTGTAATCC--3′

GLAD-F: 5′-
TAGTCCAGTGTGGTGGAATTCCACCATGCGCCTAAAAGAGACCACAAAAAAGTCGAC-3′

GLAD-R: 5′- ATCATCCTTGTAATCGCTGGAGCTGCACACGAGC-3′

AttA-F: 5′-GATCTGCGGCCGCGGCTCGAGcaccatgCAGAACACAAGCATCCTA-3′

AttA-R: 5′-CCTTCACAAAGATCCTCTAGAttaAGCGTAATCTGGAACGTCATAT-3′

Generation of the *GLAD*-HA$^{\text{knock-in (KI)}}$ flies

The *GLAD*-HA^{KI} fly strain was generated using the CRISPR/Cas9 system with two gRNAs and one recombination plasmid, as briefly summarized below.

Construction of the pCR2-TOPO-GLAD-2xHA-PBac-3xp3-eGFP HR donor vector: The L-arm of the *GLAD* flanking sequence without the stop codon was generated by PCR of the genomic DNA from the w^{1118} flies with the following primers:

F：5′-atccactagtgctagcTCAGACGGAGGCCGTAAATG-3′

R：5′-aacatcgtatgggtaGCTGGAGCTGCACACGAGC-3′. A PCR product of 1237 bp was generated and subcloned into the pCR2-TOPO-2xHA-PBac-3xp3-eGFP plasmid (a gift from Dr. C.-H. Chen) using the Nhe I and Bbs I sites to generate an intermediate vector. The R-arm of the *GLAD* flanking sequence was generated using the following primers:

F：5′-AGACGCATATGATCGCTCGAGCGCAAGGCATGAGCCATTGC-3′

R：5′-GGGCGAATTGGGCCCTCTAGAGGCCATCAATGGGTTAAGCCTG-3′.

A PCR product of 1274 bp was generated and cloned into the above intermediate vector using the Xho I and Xba I sites to generate the complete pCR2-TOPO-*GLAD*-2xHA-PBac-3xp3-eGFP HR donor vector.

To avoid the cleavage of the pCR2-TOPO-*GLAD*-2×HA-PBac-3×p3-eGFP HR donor vector by gRNA *in vivo*, we incorporated silent mutations in the homology arms (SMHA) using the Mut Express II Fast Mutagenesis Kit V2 (Vazyme) with the following primers:

SMHA-F: 5′-AACTCATAGGCTCGGCGaCAGAACATCACCAGCTCCGGATTGG-3′

SMHA-R: 5′-CGCCGAGCCTATGAGTTTCGGACGCGACGTCTGCTCAA-3′

Construction of the U6.3-GLAD-stop gRNA vector: Two CRISPR/Cas9 target sites close to the stop codon of *GLAD* were predicted using the flyCRISPR Optimal Target Finder platform:

gRNA-1: 5′-TCCGGAACTCATAGGCTCGG-3′

gRNA-2: 5′-TTGAGCAGACGTCGCGTC-3′

The fragments of the gRNA target sites were generated by PCR and subcloned into the U6b vector (a gift from Dr. R. Jiao) using the Bbs I sites to generate the U6b-*GLAD*-stop gRNA-1 and gRNA-2 plasmids.

Embryo injection and transformant selection: The two U6b-*GLAD*-stop gRNA-1 and gRNA-2 plasmids (50 ng/μL each) were mixed with the pCR2-TOPO-*GLAD*-2×HA-PBac-3×p3-eGFP HR donor vector (250 ng/μL) and injected into the *nos-Cas9* fly embryos. Successful KI flies were identified following GFP and confirmed by PCR.

RNA extraction and real-time quantitative PCR (qPCR)

For quantitative PCR (qPCR), total RNA was isolated from fly heads or cell cultures using TRIzol (Invitrogen, 15596018) according to the manufacturer's instruction. After DNase (Promega, M6101) treatment to remove genomic DNA, the RT reactions were performed using Reverse Transcriptase M-MLV (RNase H-) (Takara, #2641A). The cDNA was then used for real-time qPCR using the SYBR Select Master Mix (Life Technologies, 4472908) with the QuantStudio™ 6 Flex Real-Time PCR system (Life Technologies). The mRNA levels of *dActin5C* were used as an internal control to normalize the mRNA levels of genes of interest. The qPCR primers used in this study are listed below:

dActin5C-F: 5′-GAGCGCGGTTACTCTTTCAC-3′

dActin5C-R: 5′-GCCATCTCCTGCTCAAAGTC-3′

GLAD-RA/E/I-F: 5′-CAGTGGCCGTGCCCAATACG-3′

GLAD-RA/E/I-R: 5′-TCGACCCGCCTCCCAAAGTG-3′

GLAD-RD/F/G-F: 5′-TCATTTTCGCGACATTTTGT-3′

GLAD-RD/F/G-R: 5′-GCGATATCAGCGGTCCAG-3′

GLAD-all-F: 5′-GCACCTCTGGCAGAATCC-3′

GLAD-all-R: 5′-GGTATGCTCGGGTCGGTAG-3′

PGRP-SD-F: 5′-TTCTCGGACATTGGCTAC-3′

PGRP-SD-R: 5′-GAACCATCGTTATTGGGAC-3′

Rel-F: 5′-GCAGTTCTGGAGCAAAGT-3′

Rel-R: 5′-CCAATTCCAAGGGAGTATG-3′

AttA-F: 5′-ACAATGTGGTGGGTCAGGT-3′

AttA-R: 5′-GTGTTTTGGTCAAAGAGGC-3′

Drs-F: 5′-CTCTTCGCTGTCCTGATGC-3′

Drs-R: 5′-GGCACAGGGACCCTTGTAT-3′

TotA-F: 5′-GGTTTGCTTCAGCGTTCCA-3′

TotA-R: 5′-TGTCAGCCTCACGATCTTCG-3′

GstD1-F: 5′-CCTTACCGTAGCCGACATT-3′

GstD1-R: 5′-TCACCTTCTTGGCGTTCT-3′

GstD2-F: 5′-ACGGATTCTCCATCTGGG-3′

GstD2-R: 5′-TGATCACGGCACGCTTCT-3′

GstD3-F: 5′-TTCTCGCCAATGTCTCCA-3′

GstD3-R: 5′-TCCTGCCCAGTTTTCTTCC-3′

Cyp4e3-F: 5′-GGCGATACTGCGATAAGGA-3′

Cyp4e3-R: 5′-GGACGAAAATGATGGGGAT-3′

Cyp4s3-F: 5′-CCTGGTCAACGAGAAGCAA-3′

Cyp4s3-R: 5′-CAACTCCAACATGGCAAACT-3′

RNA-seq and data analysis

Total RNA from 100 fly brains of each genotype per duplicate was extracted using TRIzol (Invitrogen, 15596018) according to the manufacturer's instruction. Quality control and sequencing was done by Novogene Bioinformatics Technology Co. Ltd. A total amount of 3 μg RNA per sample was used as input material for the RNA sample preparations. Sequencing libraries were generated using NEBNext® UltraTM RNA library Prep Kit for Illumina® (NEB, USA) following manufacturer's recommendations and index codes were added to attribute sequences

to each sample. Paired-end RNA-seq libraries were prepared following Illumina's protocols and sequenced 150 bp paired-end on the Illumina HiSeq 4000 platform. We aligned the reads to the *Drosophila melanogaster* reference genome (dm6) using STAR v2.5.2b. Gene expression levels were measured using read counts. Differentially expressed genes (DEGs) were calculated using DESeq with the cutoff as follows: p value < 0.05 and fold change > 1.5. Gene Ontology (GO) enrichment analysis of differentially expressed genes was implemented by DAVID Bioinformatics resources 6.8 (Huang et al., 2009) and GO category (Ashburner et al., 2000). GO terms with corrected p value (Benjamini) less than 0.05 were considered significantly enriched by differential expressed genes.

Paraffin sections and vacuole measurement

Adult male fly heads were fixed in Bouin's solution for 4-7 days, and then embedded in the paraffin and sectioned at 8 μm thickness. Neurodegeneration was assessed by quantification of vacuoles in the fly brain. All vacuoles in each section were counted. At least five fly brains were analyzed for each genotype. The experiment was repeated more than three times.

Immunohistochemistry

Whole-mount adult fly brains or third instar larval salivary gland cells were dissected and immunoblotted according to standard protocols. The following antibodies were used: mouse anti-repo, rat anti-elav and mouse anti-HP1a (DSHB, 1:100); rabbit anti-HA (CST, 1:100), rabbit anti-H3K27me3 (Abcam, 1:1000); rat anti-HA (Roche, 1:500). Secondary antibodies were coupled to Alexa532, Alexa568 or Cy5 (Invitrogen, 1:500). Images were taken on Leica TCS SP8 with the pulsed White Light Laser (WLL) or Leica TCS SP8 and analyzed with Leica Application Suite X (LAS X) software and images were further processed using Adobe Photoshop 6. Every experiment was performed more than three times.

Antibodies

The following antibodies were used for Western blotting and immunofluorescence assays: Mouse anti-GFP (Abmart, 7G9), rabbit anti-tubulin (MBL, PM054), mouse anti-repo (DSHB, 8D12), rat anti-elav (DSHB, 7E8A10), rabbit anti-HA (CST, C29F4), rat anti-HA (Roche, 11867423001), rabbit anti-H3K27me3 (Abcam, ab192985), mouse anti-HP1a (DSHB, C1A9), mouse anti-FLAG (Sigma-Aldrich, F1804). HRP conjugated secondary antibodies: anti-mouse (Sigma-Aldrich, A4416), anti-rabbit (Sigma-Aldrich, A9169), and anti-rat (Sigma-Aldrich, A9037). Fluorescent secondary antibodies: anti-rabbit Alexa Fluor® 532 (Life Technologies, A11009), anti-mouse Alexa Fluor® 568 (Life Technologies, A11031), anti-rat Alexa Fluor® Cy5 (Life Technologies, A10525), anti-rabbit Alexa Fluor® 488 (Life Technologies, A11034).

Protein extraction and Western blotting

Fly heads were homogenized and lysed in 2X LDS sample loading buffer (ThermoFisher # NP0007) containing protease and phosphatase inhibitor cocktails (Roche, 04693132001). Samples were sonicated, boiled for 5 minutes and then centrifuged at 12,000 g for 10 min at 4°C. The supernatants were then loaded in 3-8% NuPAGE tris-acetate gels (Invitrogen) and probed with the primary and secondary antibodies listed above. The immunoblots were detected using the High-sig ECL Western Blotting Substrate (Tanon). Images were captured with an Amersham Imager 600 (GE Healthcare) and the densitometry was measured with ImageJ. The contrast and brightness were adjusted equally using Adobe Photoshop CC2019. Tubulin or GAPDH was used as a loading control.

In vivo BBB integrity assay

The fly BBB integrity was assessed by an *in vivo* assay as previously described. Briefly, ether-anesthetized adult male flies were injected with thin borosilicate needles containing 12.5 mg/mL 10 kDa Dextran Texas Red® (Invitrogen, D1863) and left to recover overnight. Fly heads were

fixed *in situ* for 15 min with 4% paraformaldehyde prior to brain dissection. And then brains were dissected in PBS and mounted in anti-fade mounting medium (Vectashield, H-1000). For an intact fly BBB, the Texas Red-labeled dextran (10 kDa) injected into the fly body would circulate within the hemolymph and not penetrate into the brain. When the BBB integrity was disrupted, the Dextran dye would leak through and Texas Red would be detected inside the fly brain.

Cell culture and transfection

Drosophila S2R+ cells were cultured in Schneider's *Drosophila* Medium (ThermoFisher # 21720001) supplemented with 10% FBS (Vistech, SE200-ES). Expression plasmids were transfected using the X-tremeGENE™ HP Transfection Reagent (Roche, 6366236001) following the manufacturer's instructions, and the cells were collected 48 h after transfection.

ChIP-qPCR

S2R+ cells transfected with pIZ-3xFLAG or pIZ-*GLAD*-3xFLAG were cross-linked in 1% vol/vol formaldehyde/PBS for 10 min at room temperature and then stopped with Glycine (125 mM) for 5 min at room temperature. Samples were lysed in RIPA buffer (50 mM Tris-HCL, 150 mM sodium chloride, 1.0% NP-40, 0.5% sodium deoxycholate, 0.1% sodium dodecyl sulfate, 1 mM EDTA, 1 mM EGTA, pH 7.8) at 4°C for 2 h and then homogenized using a Bioruptor® Plus sonication device (Diagenode, UCD-300). The supernatants were incubated with Protein A/G Dynabeads (Thermo Fisher Scientific, 26161) associated with the mouse anti-FLAG antibody (Sigma-Aldrich, F1804) or mouse IgG (Santa Cruz Biotech, SC-2025) at 4°C for 5 h. Subsequently, elution and reverse cross-link were performed at 65°C for 30 min on a thermomixer. The DNA was then isolated using the QIAquick PCR Purification Kit (QIAGEN) according to the manufacturer's instruction. The purified DNA was subjected to qPCR analysis to evaluate the occupancy by the GLAD protein at the promoter region. The qPCR primers of the ChIP-qPCR assays used in this

study are listed below:

1360-F: 5′-GGAGCTCTGCGTATAGCCAACTT-3′

1360-R: 5′-ACCTAAACCGCCGAGTCCTG-3′

H2A-H2B-F: 5′-CACGGTTTGAGCGGGACT-3′

H2A-H2B-R: 5′-TCTGGGTTAGGCGAGCAT-3′

H3-H4-F: 5′-TCACCGTCCACGATTGCT-3′

H3-H4-R: 5′-TCTCCGATTTGGGTTTCA-3′

rp49-F: 5′-CGATCTCGCCGCAGTAAAC-3′

rp49-R: 5′-CTTCATCCGCCACCAGTCG-3′

dActin5C-F: 5′-AGAGCGAAAGCCAGACGA-3′

dActin5C-R: 5′-CTATGGGTGCGAAGGAGA-3′

Rel-F: 5′-TCCCATTGTCCAGGTCGTA-3′

Rel-R: 5′-AGGCAGCGGATCACTTTT-3′

AttA-F: 5′-AAGTGGCGTCAATGGGTC-3′

AttA-R: 5′-ATGGTGGTTTACTTCTGCTGTT-3′

Drs-F: 5′-CTATTAGGCCGGATGTTT-3′

Drs-R: 5′-ATGCGTTACTCAATGAAGAT-3′

TotA-F: 5′-ATTGCGATTGCCACCAGA-3′

TotA-R: 5′-CTCCCTCACATGAAGTTAGACC-3′

GstD1-F: 5′-AAAAGTAGCACTGAACGGAATT-3′

GstD1-R: 5′-CCCAGGCACGACAACAAC-3′

GstD3-F: 5′-GCTCCGTTCTGATGGTGG-3′

GstD3-R: 5′-ATCGGGATTCATTTGCTC-3′

RNAi interference (RNAi) treatment

For RNAi downregulation in S2R+ cells, DNA templates of T7 promoter-containing *Gal4* or *GLAD*

were amplified by PCR and subcloned into pCR2.1 vector (Invitrogen). dsRNA was synthesized by using a T7 RiboMax kit (Promega) following manufacturer's protocol. A total of 1 × 10^6 cells in a 6 wells plate were incubated with 2 µg of dsRNA for 3 days. The efficiency of RNAi and the expressions of immune-related genes were checked by qPCR.

Cell cycle synchronization

Cell cycle synchronization of *Drosophila* S2R+ cells was conducted as previously described. Briefly, log-phase (1.5 x 10^6/mL) cells were first incubated with 1.7 μM Hydroxyecdysone (Selleck, S2417), 24 h to obtain G2 cells. Cells were then rinsed with phosphate-buffered saline (PBS) three times, resuspended in fresh Schneider's *Drosophil*a Medium (ThermoFisher #21720001) supplemented with 10% FBS (Vistech, SE200-ES), along with 1.5 mM Hydroxyurea (Selleck, S1896), and cultured for 18 h to obtain G1/S cells. Afterward, these cells were rinsed with PBS three times, cultured in the above-specified medium without Hydroxyurea, and harvested at various time points for FACS analyses or qPCR analyses. These cells were ethanol-fixed and then treated with propidium iodide and RNase A for 30 min before FACS analyses.

Statistical analysis

Unless otherwise noted, the statistical significance in this study is determined by log-rank test, one-way ANOVA with Tukey's HSD post-hoc test, two-way ANOVA with Bonferroni’s post-hoc test, or unpaired, two-tailed Student’s *t*-test at $^{*}p < 0.05$, $^{**}p < 0.01$, and $^{***}p < 0.001$. The error bars represent the standard error of the mean (SEM).

SUPPLEMENTAL FIGURES AND FIGURE LEGENDS

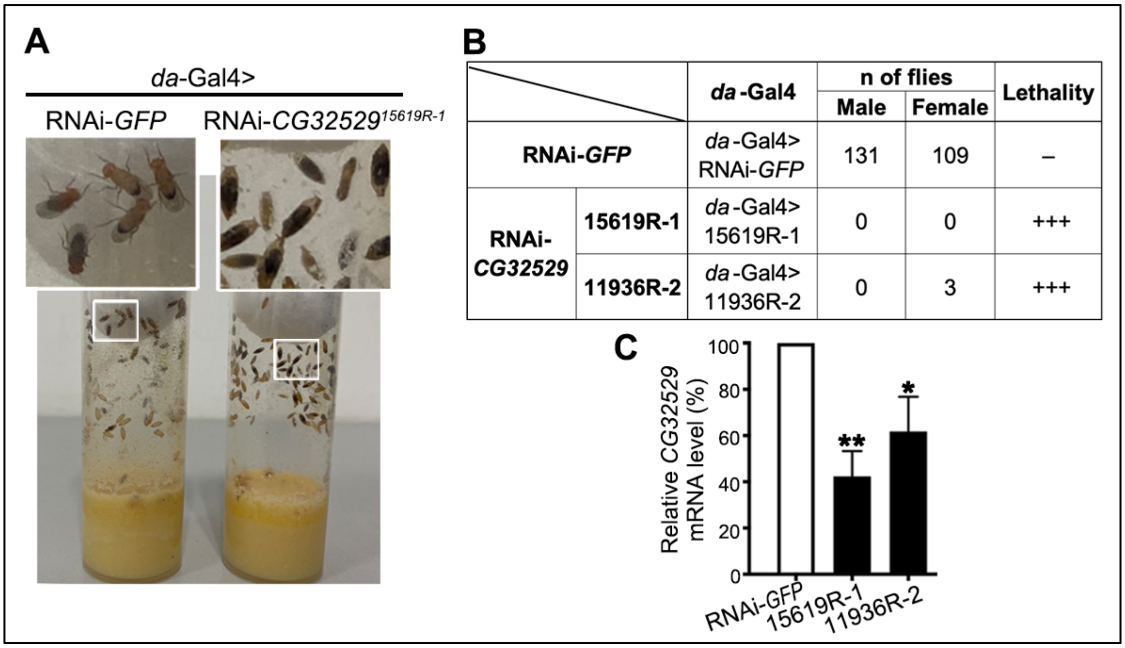

		da-Gal4	n of flies		Lethality
			Male	Female	
**RNAi-*GFP***		*da*-Gal4> RNAi-*GFP*	131	109	–
**RNAi-*CG32529***	**15619R-1**	*da*-Gal4> 15619R-1	0	0	+++
	**11936R-2**	*da*-Gal4> 11936R-2	0	3	+++

Figure S1. Ubiquitous KD of *CG32529* causes developmental lethality, related to Figure 1

(**A**) Representative images showing the control flies (*da*-Gal4>RNAi-*GFP*) that developed to adults and successfully eclosed (left) and the *da*-Gal4>*CG32529*$^{15619R-1}$ flies that died in the pupal case (right). (**B**) The numbers (n) of male and female flies that survive to the adulthood with ubiquitous KD (with *da*-Gal4) of *CG32529* by two independent RNAi lines are counted. (**C**) The mRNA levels of *CG32529* in the heads of the flies with adult-onset KD (*Tub*GS) by the two RNAi lines are examined by qPCR analysis. Data are presented as mean ± SEM, n = 3; the statistical significance is determined by one-way ANOVA with Tukey's HSD post-hoc test. *$p < 0.05$ and **$p < 0.01$. (See Table S2 for the specific genotypes of the flies tested in each figure.)

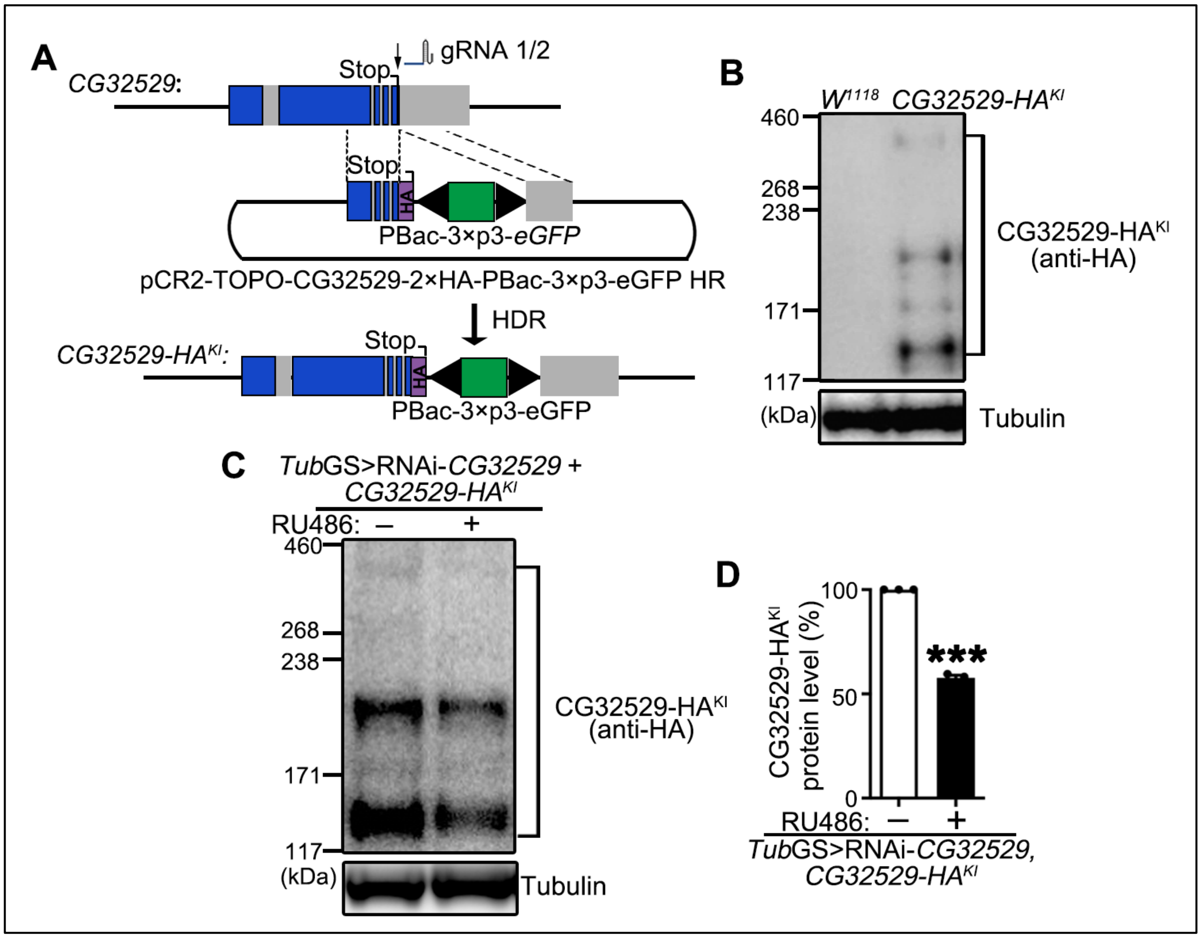

Figure S2. Generation and examination of the *CG32529-HA^KI* fly line, related to Figure 1 and 2

(**A**) A simplified diagram showing the design and process to generate the *CG32529-HA^KI* flies using the CRISPR-cas9 system (Port et al, 2016; Lin et al, 2014; see Methods for details). (**B**) Multiple bands of the CG32529-HA^KI protein isoforms are expressed in the *CG32529-HA^KI* fly heads, examined by western blotting with an anti-HA antibody. (**C-D**) Representative image (C) and quantification (D) of the western blots confirming decreased protein levels of the CG32529-HA^KI isoforms in the *Tub*GS>RNAi-*CG32529* fly heads. Mean ± SEM, n = 3; Student's *t*-test. ***$p < 0.001$.

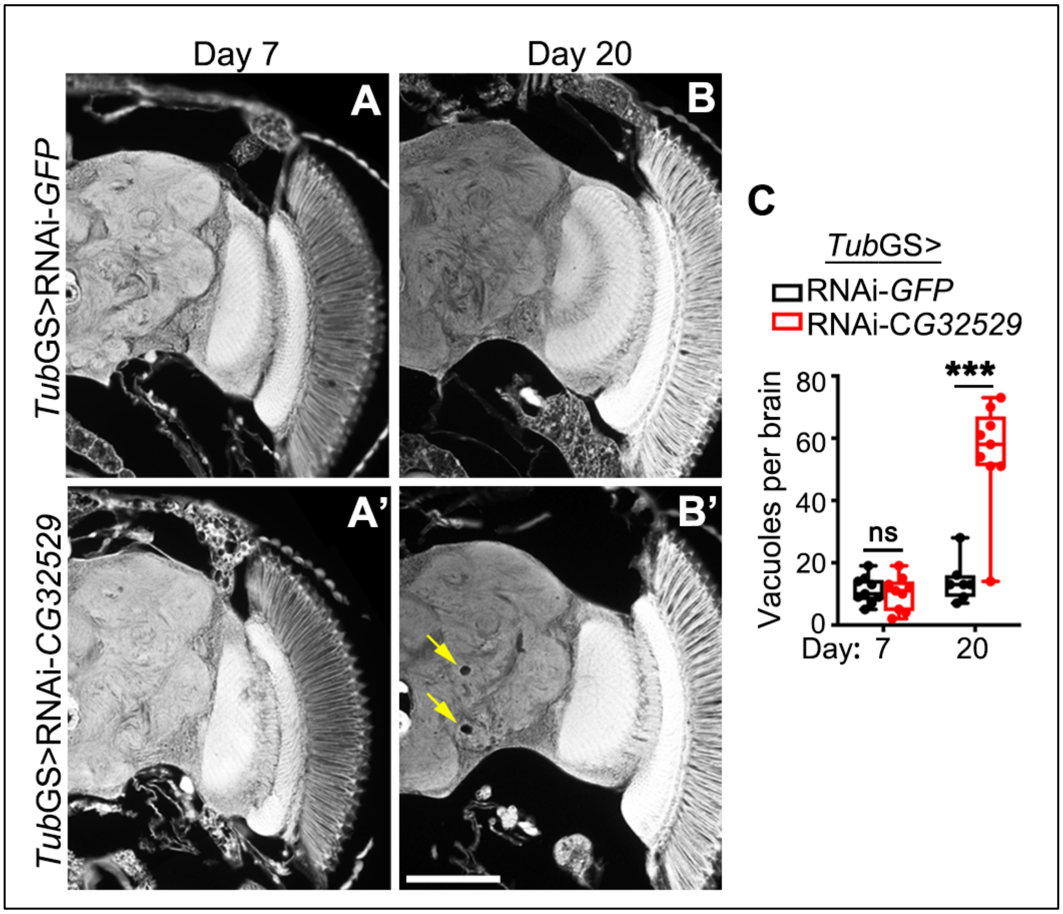

Figure S3. Adult-onset KD of the *CG32529* cause age-dependent brain degeneration, related to Figure 1 and 2

(**A-B'**) Representative images of brain paraffin sections of the *Tub*GS>RNAi-*GFP* (A-B) or *Tub*GS>RNAi-*CG32529* (A'-B') flies at the indicated ages. Arrows, brain vacuoles. Scale bar: 100 μm. (**C**) Counts of total brain vacuoles per fly brain (~30 sections/brain). Data are shown as boxplot, n = 7-9 fly brains each group; Student's *t*-test. ***$p < 0.001$; ns, not significant.

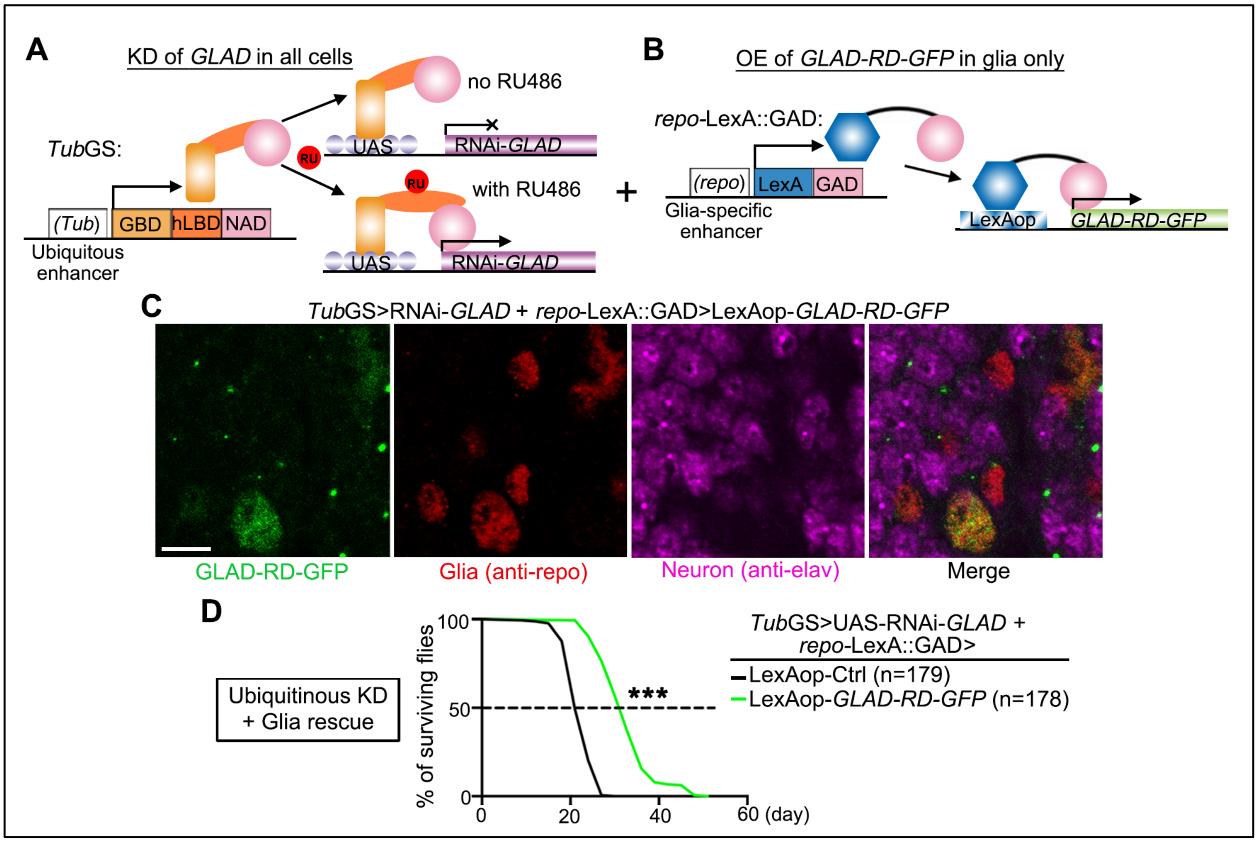

Figure S4. Glia-specific rescue of the ubiquitously downregulated *GLAD* using the dual binary expression systems, related to Figure 2

(**A-B**) A schematic illustration of the dual binary expression systems of the GS/UAS (Ostervalder et al., 2001; Roman et al., 2001) (A) and the LexA::GAD/LexAop (Lai and Lee, 2006) (B). In brief, the ubiquitously expressed *Tub*GS activates the transcription of the UAS-RNAi-*GLAD* transgene in the presence of RU486, a ligand of human progesterone receptor ligand binding domain (hLBD). Meanwhile, we generate a LexAop-*CG32529-RD*-GFP fly line and use the *repo*-LexA::GAD to drive its expression in glia specifically. Because the two binary expression systems are independent of each other, we are able to downregulate *GLAD* in all cells while rescue the KD only in glia in the "*Tub*GS>RNAi-*GLAD* + *repo*-LexA::GAD>LexAop-*GLAD-RD-GFP*" flies (examined in C-D). GBD, Gal4 DNA binding domain; NAD, NF-kB activating domain; GAD, Gal4 DNA activating domain. (**C**) Representative confocal images of whole-mount fly brains confirming

the specific expression of LexAop-*GLAD-RD-GFP* with the *repo*-LexA::GAD driver in glia (anti-repo) but not neurons (anti-elav) of the *Tub*GS>RNAi-*GLAD* flies. Scale bar: 5 μm. (**D**) The lifespan of the *Tub*GS>RNAi-*GLAD* flies is partially rescued by glia-specific OE of *GLAD-RD*-GFP. LexAop-Ctrl, LexAop-*Luciferase*. Log-rank test, the number (n) of flies in each group is as indicated. ***$p < 0.001$.

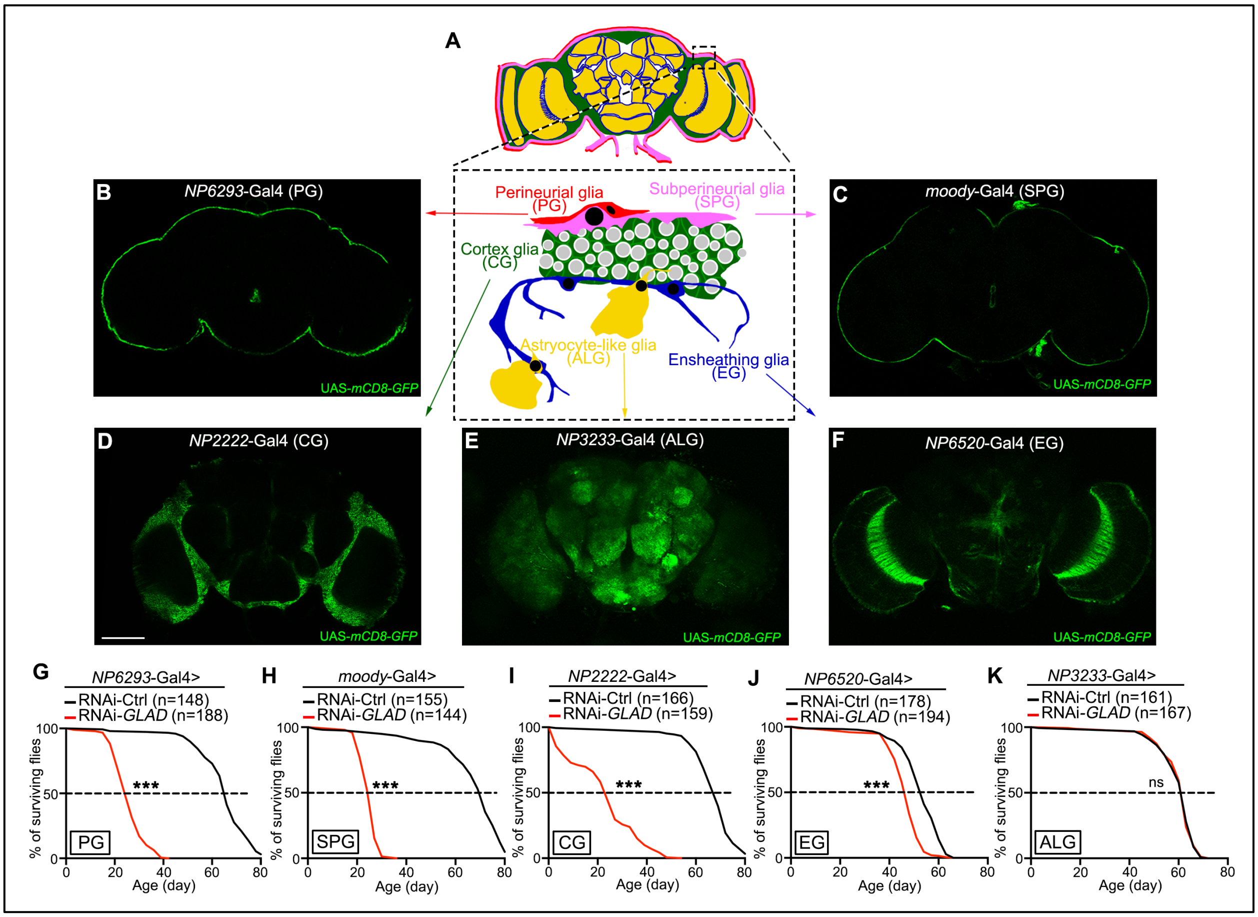

Figure S5. The function of *GLAD* shows glial subtype-specificity, related to Figure 2 and 3

(**A**) A schematic cartoon and zoom-in of the boxed area illustrating the distribution and morphology of the five subtypes of glia in the fly brain [adapted from Kremer et al., 2017]. (**B-F**) Representative confocal images of the whole-mount fly brains confirming the expression patterns of UAS-*mCD8-GFP* driven by the indicated glial subtype Gal4 drivers. PG (B), perineurial glia (*NP6293*-Gal4) and SPG (C), subperineurial glia (*moody*-Gal4), which are the surface glia forming the fly BBB; CG (D), cortex glia (*NP2222*-Gal4), which wrap individual neuronal cell body; ALG (E), astrocyte-like glia (*NP3233*-Gal4), which penetrate deeply into the neuropil and strongly associated with synapses; EG (F), ensheathing glia (*NP6520*-Gal4), which wrap axons and divide the neuropils. Scale bar: 100 μm. (**G-K**) The lifespan assays of the flies with KD of *GLAD* in the indicated glial subtypes: PG (G), SPG (H), CG (I), EG (J) and ALG (K). RNAi-Ctrl, RNAi-*GFP*. The number (n) of flies tested in each group is as indicated; log-rank test. ***$p < 0.001$; ns, not significant.

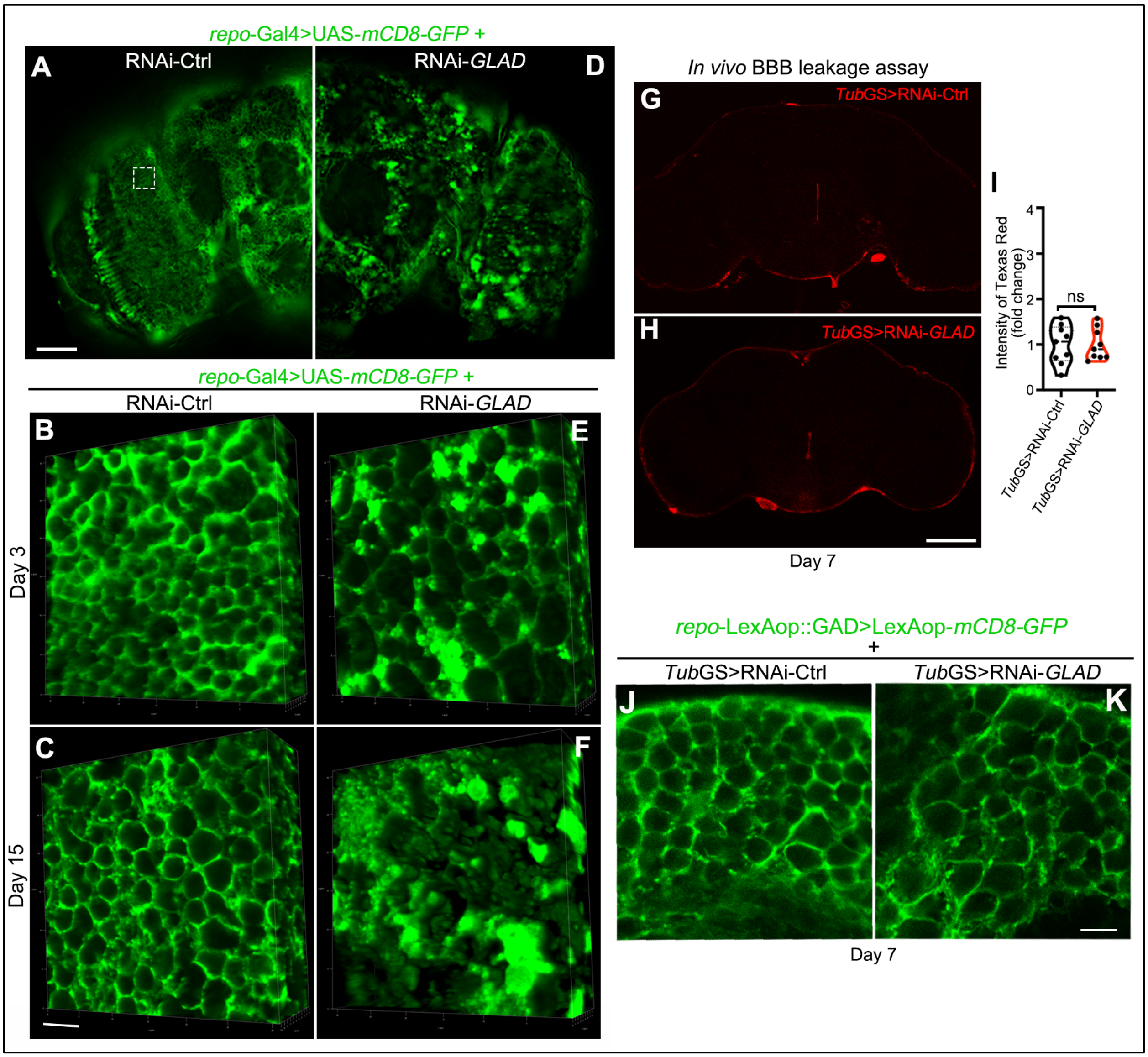

Figure S6. Examination of glia and BBB integrity of the RNAi-*GLAD* flies with *repo*-Gal4 or *Tub*GS, related to Figure 3

(**A-F**) Representative confocal images of the glial meshwork of the whole-mount fly brains labelled by *repo*-Gal4>UAS-*mCD8-GFP* in the RNAi-Ctrl (A-C) or RNAi-*GLAD* flies (D-E). The white box in (A) indicates the zoom-in areas shown in (B-C and E-F). The fly brains were examined on Day 3 or Day 15 and shown with 3D reconstruction (also see Supplemental Video S1 and S2). RNAi-Ctrl, RNAi-*Luciferase*. (**G-I**) Representative images of the *in vivo* BBB leakage assay (G-H) and quantification (I) of the relative intensity of Texas Red-labeled 10 kDa dextran in the brain of the

*Tub*GS>RNAi-Ctrl (RNAi-*GFP*) flies or the *Tub*GS>RNAi-*GLAD* flies on Day 7. Violin plots with each data point are shown, n = 9 fly brains each group. Student *t*-test. ns, not significant. (**J-K**) Representative confocal images confirming that the glial meshwork of the *Tub*GS>RNAi-*GLAD* flies on Day 7 displays normal morphology and no marked glial deformation is detected. Of note, the commonly used fly glial (repo) and neuronal (elav) markers are both expressed in the nucleus and cannot be used to characterize the cell morphology of fly glia or neurons. Instead, *in vivo* imaging of genetically-encoded, plasm membrane-bound fluorescent protein (e.g., mCD8-GFP) is required, which involves the dual binary systems to genetically KD *GLAD* in glia and *in vivo* image glial cell morphology at the same time but independently. Here, glia in the *Tub*GS>RNAi-*GLAD* flies are labelled specifically and independently by *repo*-LexAop::GAD>LexAop-*mCD8-GFP*. Scale bars: 50 μm in (A and D), 5 μm in (B-C, E-F and J-K), and 100 μm in (G-H).

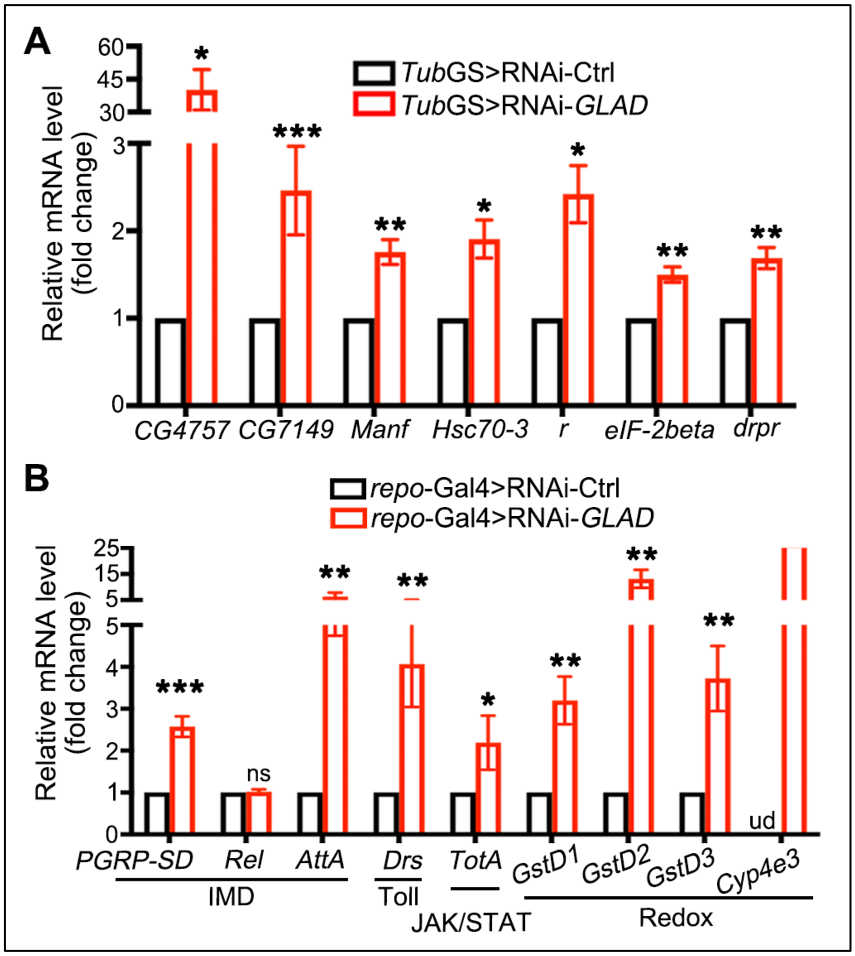

Figure S7. Additional validations related to the RNA-seq analysis of the RNAi-*GLAD* flies, related to Figure 4

(**A**) The qPCR analysis confirming the upregulation of additional, non-immune related DEGs in the heads of the *Tub*GS>RNAi-*GLAD* flies. (**B**) The qPCR analysis of the mRNA levels of immune and related genes in the heads of the *repo*-Gal4>RNAi-*GLAD* flies. The mRNA levels of each gene are normalized to *actin* and set to 1 in the RNAi-*GFP* (RNAi-Ctrl) flies, and the relative mRNA levels in the RNAi-*GLAD* flies are shown as fold change. Mean ± SEM; n = 3-4 in (A) and n = 4 in (B). Student's *t*-test. **p* < 0.05, ***p* < 0.01, ****p* < 0.001; ns, not significant. ud, undetected. Of note, because the basal level of *Cyp4e3* in the control flies is too low to be detected in the qPCR analysis, the increase of its expression in the *repo*-Gal4>RNAi-*GLAD* flies is shown as an infinite fold change.

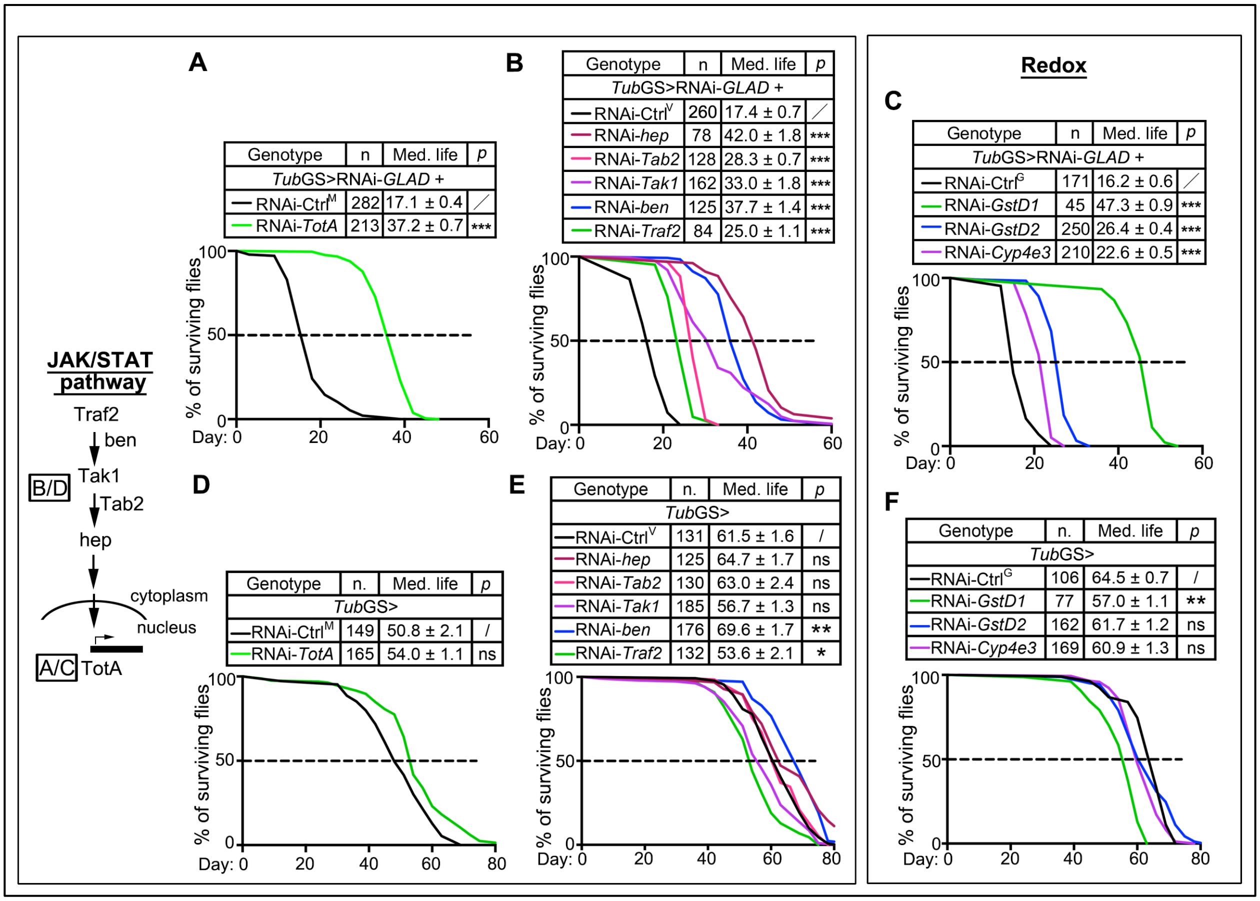

Figure S8. The impact of adult-onset downregulation of the JAK/STAT pathway or redox genes in the RNAi-*GLAD* or WT flies on longevity, related to Figure 5

(**A-C**) The lifespan rescue experiments by adult-onset downregulation of the JAK/STAT pathway (A-B) or redox (C) genes in the *Tub*GS>RNAi-*GLAD* flies. (**D-F**) The lifespan experiments of the flies with adult-onset, ubiquitous KD (*Tub*GS) of the above JAK/STAT pathway (D-E) or redox (F) genes in WT flies without RNAi-*GLAD*. The specific backcrossed isogenized RNAi control lines are used according to the origin: $Ctrl^M$, RNAi-*mCherry* (BDSC, THFC); $Ctrl^V$, RNAi-60200 (VDRC); $Ctrl^G$, RNAi-*GFP* (NIG-FLY). n, number of flies tested for each genotype. Med. life, median lifespan, calculated as mean ± SEM of days when 50% or more flies in a vial have died. *p*, statistical significance of the median lifespans, determined by one-way ANOVA. **p* < 0.05, ***p* < 0.01, ****p* < 0.001; ns, not significant.

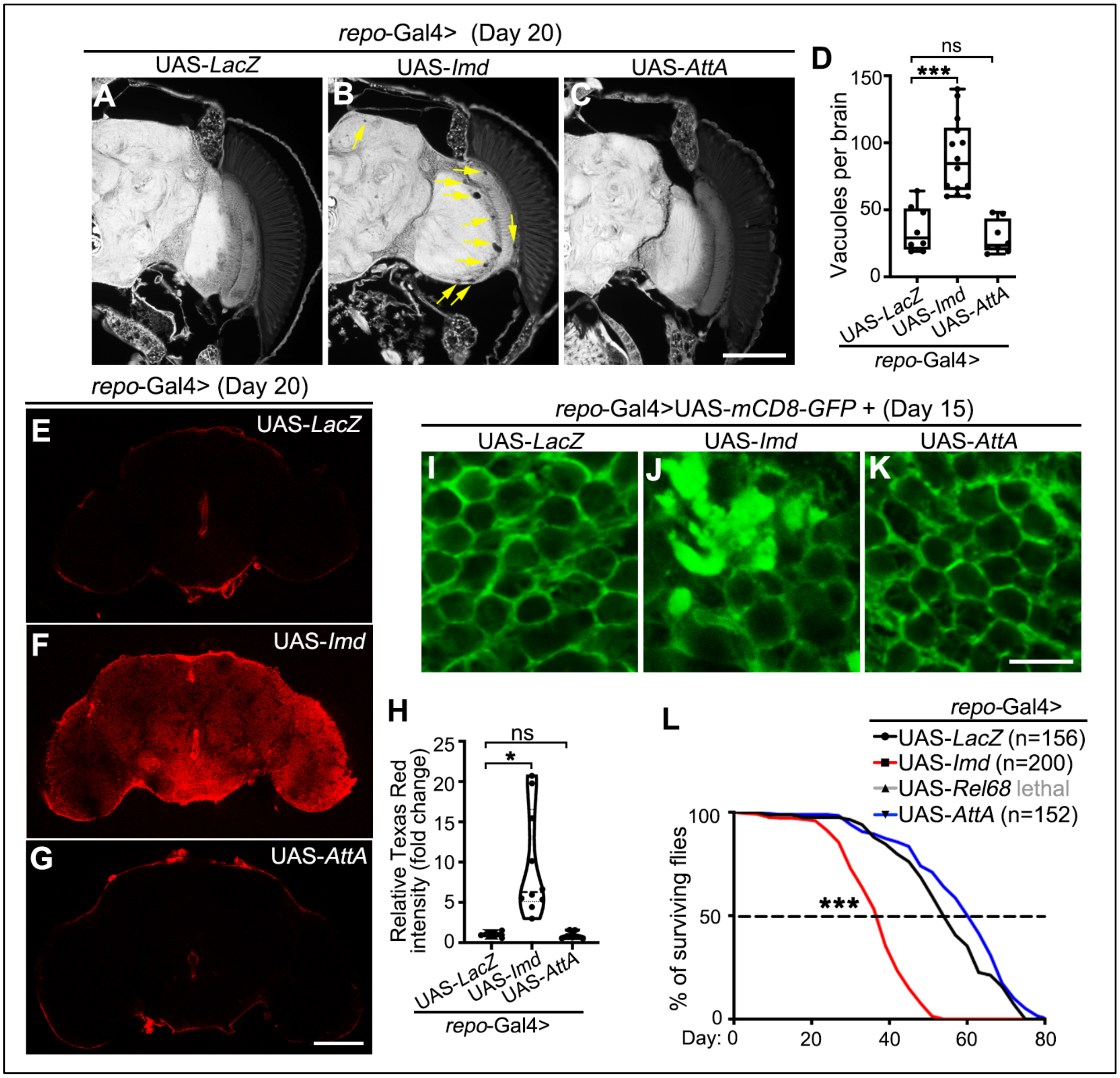

Figure S9. OE of *Imd* in glia is sufficient to disrupt BBB and glial integrity and shorten the lifespan in flies, related to Figure 5

(**A-D**) Representative images (A-C) and quantification (D) of the brain vacuoles in the paraffin sections of the flies with glial OE of *LacZ* (control) (A), *Imd* (B) or *AttA* (C) on Day 20. Arrows, brain vacuoles. (**E-H**) Representative images (E-G) and quantification (H) of the BBB leakage assay of the flies with glial OE of *LacZ* (E), *Imd* (F) or *AttA* (G) on Day 20. (**I-K**) Representative images of the glial meshwork in the fly brain with glial OE of *LacZ* (I), *Imd* (J) or *AttA* (K) on Day

15. (**L**) The lifespan assay of the flies of the indicated genotypes. Note that OE of the constitutive Relish (*Rel68*) in glia is lethal and thus cannot be examined in the above fly brain or lifespan assay. n = 8-14 in (D), n = 6-10 in (H), and the number (n) of flies tested in each group is as indicated in (L). One-way ANOVA in (D, H) and log-rank test in (L). *$p < 0.05$, ***$p < 0.001$; ns, not significant. Scale bars: 100 μm in (A-C, E-G) and 5 μm in (I-K).

A

	Actin-Gal4/+	n		Lethality
		male	female	
UAS-*LacZ*	*Actin*-Gal4/ UAS-*LacZ*	72	72	-
	+/ UAS-LacZ	76	64	
UAS-*GLAD*	*Actin*-Gal4/ UAS-*GLAD*	0	0	+++
	+ / UAS-*GLAD*	57	80	

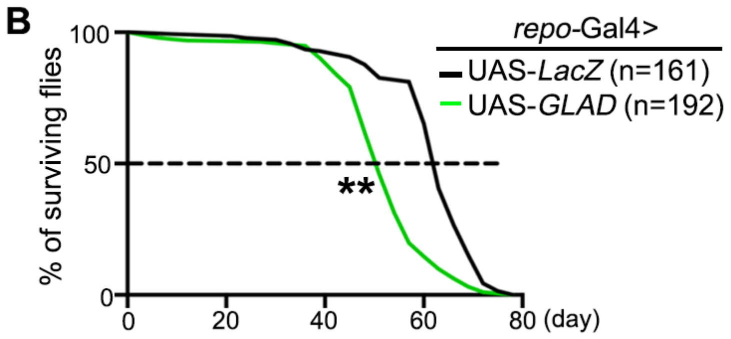

Figure S10. OE of *GLAD* causes developmental lethality and shortens the lifespan, **related to Figure 4 and 5**

(**A**) The number (n) of male and female flies that survive to the adulthood with ubiquitous OE of *GLAD* or the control gene *lacZ* (with or without the *Actin*-Gal4 driver) from each cross is shown. (**B**) Upregulation of *GLAD* in glia (*repo*-Gal4) shortens the lifespan. Log-rank test, the number (n) of flies in each group is as indicated. ***p* < 0.01.

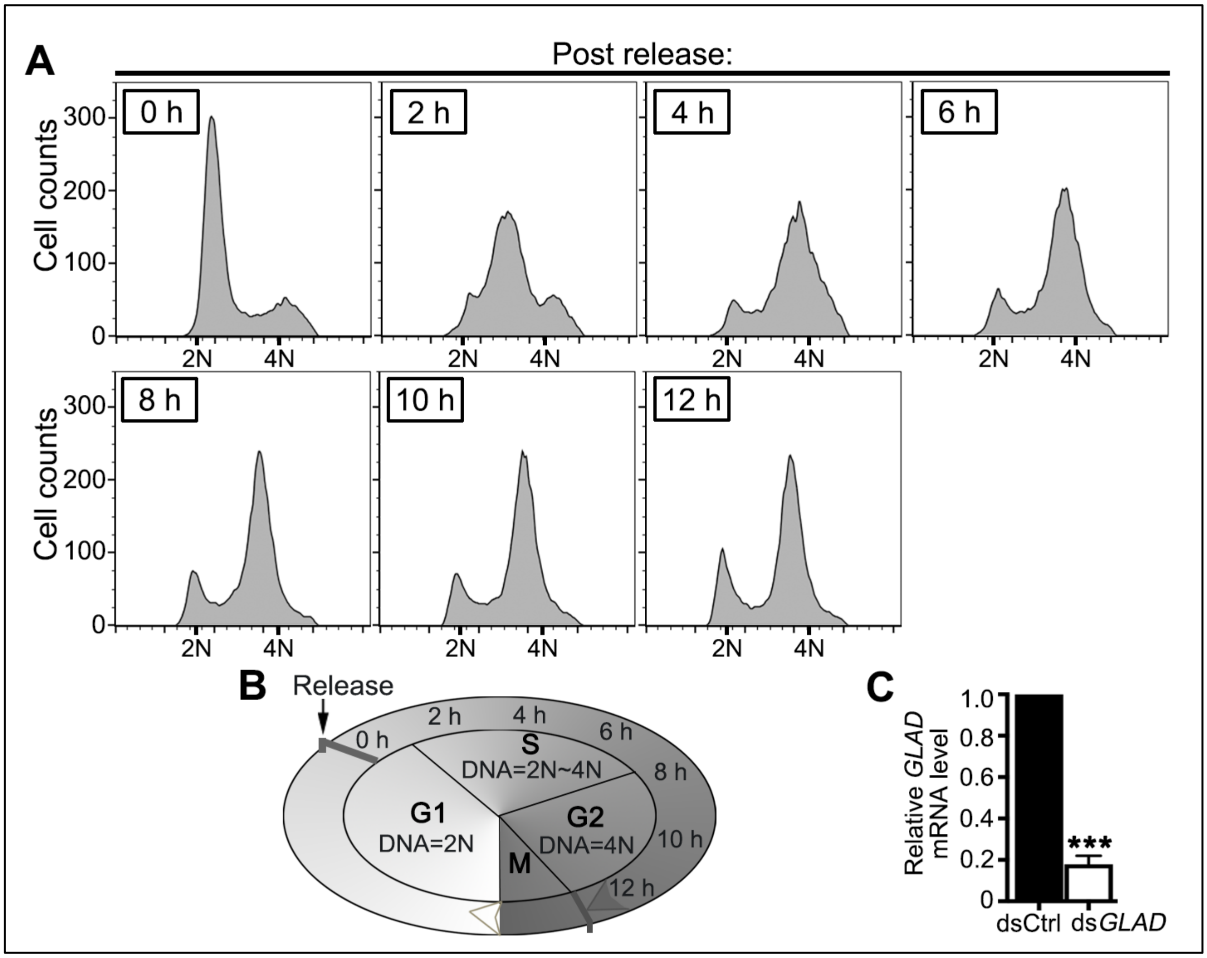

Figure S11. Validation of the different phases and the KD efficiency of ds*GLAD* in the cell cycle assay, related to Figure 6

(**A**) The different phases in the cycle of cell division are determined by the flow cytometry analysis. Briefly, *in vitro* cultured *Drosophila* S2R+ cells are synchronized and ceased at the G1 phase (DNA = 2N) using hydroxyurea (1.5 mM). (**B**) Removal of hydroxyurea releases the cells from the G1 phase to the S phase (DNA = 2N to 4N) in 2-6 h, and then to the G2/M phase (DNA = 4N) in 8-12 h. G1 phase, growth and metabolism; S phase, DNA replication; G2 phase, growth of structural elements; M phase, mitosis. (**C**) The KD efficiency of ds*GLAD* in S2R+ cells is examined by qPCR. dsCtrl, scrambled dsRNA; ds*GLAD*, dsRNA against *GLAD*. Mean ± SEM, n = 5; Student's *t*-test. ***$p < 0.001$.

SUPPLEMENTAL TABLES

S**upplemental Table S1. The RNA-seq results of the 466 DEGs of the TubGS>RNAi-GLAD flies, related to Figure 4**

Supplemental Table S2. A summary of the genotypes of the flies examined in each figure, related to Figure 1-6 and Figure S1-S10

SUPPLEMENTAL VIDEOS

Supplemental Video S1. Representative 3D reconstruction of the glial meshwork (*in vivo* labeled by UAS-*mCD8-GFP*) of the *repo*-Gal4>RNAi-Ctrl flies on Day 15, related to Figure 3 and Figure S6

Supplemental Video S2. Representative 3D reconstruction of the glial meshwork (in vivo labeled by UAS-mCD8-GFP) of the repo-Gal4>RNAi-GLAD flies on Day 15, related to Figure 3 and Figure S6

SUPPLEMENTAL REFRENCES

Ashburner, M., Ball, C.A., Blake, J.A., Botstein, D., Butler, H., Cherry, J.M., Davis, A.P., Dolinski, K., Dwight, S.S., Eppig, J.T., et al., 2000. Gene ontology: tool for the unification of biology. The Gene Ontology Consortium. Nat Genet 25, 25-29.

Huang, D.W., Sherman, B.T., and Lempicki, R.A., 2009. Bioinformatics enrichment tools: paths toward the comprehensive functional analysis of large gene lists. Nucleic Acids Res. 37, 1-13

Lai, S.L., and Lee, T., 2006. Genetic mosaic with dual binary transcriptional systems in Drosophila. Nat. Neurosci. 9, 703-709.

Lin, S.C., Chang, Y.Y., and Chan, C.C., 2014. Strategies for gene disruption in Drosophila. Cell Biosci. 4, 63.

Osterwalder, T., Yoon, K.S., White, B.H., and Keshishian, H., 2001. A conditional tissue-specific transgene expression system using inducible GAL4. Proc. Natl. Acad. Sci. USA 98, 12596-12601.

Port, F., and Bullock, S.L., 2016. Creating Heritable Mutations in Drosophila with CRISPR-Cas9. Methods Mol. Biol. 1478, 145-160.

Roman, G., Endo, K., Zong, L., and Davis, R.L., 2001. P[Switch], a system for spatial and temporal control of gene expression in Drosophila melanogaster. Proc. Natl. Acad. Sci. USA. 98, 12602-12607.
